# Spatial pattern assessment of *Aedes* mosquito bite risk in a subtropical metropolitan area: A case study in Shenzhen

**DOI:** 10.1371/journal.pntd.0013843

**Published:** 2025-12-23

**Authors:** Liangqiang Lin, Yifei He, Xu Guang, Lanbin Xiang, Dongfeng Kong, Kaichuan Diao, Haidong Wang, Bin Zhu

**Affiliations:** 1 Shenzhen Center for Disease Control and Prevention, Shenzhen, China; 2 School of Public Health and Emergency Management, Southern University of Science and Technology, Shenzhen, China; 3 Shenzhen Center for Chronic Disease Control, Shenzhen, China; Egerton University, KENYA

## Abstract

Climate change and urbanization are contributing to the expansion of *Aedes* mosquito populations in subtropical metropolitan areas, thereby increasing the potential for human–vector contact and associated transmission risk. As vectors of multiple pathogenic viruses, *Aedes* mosquitoes not only elevate public health risks but also impose considerable burdens on urban health systems. Beyond vector distribution, demographic characteristics and the associated spatial patterns also influence the transmission risk of diseases such as dengue fever. However, a comprehensive framework for spatially assessing *Aedes* mosquito bite risk from multiple dimensions is still lacking in subtropical regions, which hinders the formulation of targeted public health strategies. Based on the hazard-exposure-vulnerability risk assessment framework, this study systematically evaluated the *Aedes* mosquito bite risk in Shenzhen. The hazard layer was generated using the Optimal Parameters-based Geographical Detector (OPGD) and the Geographically Weighted Random Forest (GWRF) model to predict spatial patterns of *Aedes* mosquito density from surveillance data. The exposure layer was derived from population density data, while the vulnerability layer was constructed by applying Geographically Weighted Principal Component Analysis (GWPCA) to multiple demographic and socioeconomic variables. By combining hazard, exposure, and vulnerability layers, we generated a comprehensive *Aedes* mosquito bite risk map and identified high risk subdistricts in Shenzhen. Further analysis revealed the underlying drivers of each risk hotspot, enabling the proposal of tailored recommendations for *Aedes* mosquito bite risk prevention. These findings provided valuable insights for the development of preventive measures against vector-borne disease spread and offer a method that can be easily applied to other subtropical megacities.

## Author summary

*Aedes* mosquitoes transmit dengue, Zika, and chikungunya viruses, causing increasing disease burdens in rapidly urbanizing subtropical cities. Existing assessments commonly map *Aedes* mosquito occurrence but rarely integrate ecological hazard with spatial patterns of human presence and socioeconomic vulnerability. Conducted in the subtropical city of Shenzhen, China, this study developed an effective risk assessment framework to assess *Aedes* mosquito bite risk. We constructed a spatial assessment framework that integrates three key dimensions: *Aedes* mosquito hazard (where *Aedes* mosquitoes present), human exposure (where populations reside), and population vulnerability (who is most susceptible based on demographic and socioeconomic conditions). We identified the subdistricts with relatively high *Aedes* mosquito bite risk and determined the key driving factors contributing to these elevated risks. This integrated framework enhances the precision of intervention measures by identifying areas with high *Aedes* mosquito density and vulnerable social structures. It is designed to support evidence-based decision making for public health planners and urban policymakers, and can be applied to other densely populated subtropical cities to guide targeted vector control and optimize resource allocation.

## 1. Introduction

In March 2022, the World Health Organization (WHO) launched the Global Arbovirus Initiative [1] in response to the growing global challenge posed by arboviruses. This concern is underscored by the rising incidence of mosquito-borne diseases, including dengue fever, observed in many regions over recent years [2]. *Aedes albopictus* and *Aedes aegypti* are the primary vectors for mosquito-borne diseases, with a widespread presence across tropical, subtropical, and temperate regions [3,4]. Consequently, assessing the risk of *Aedes* mosquito bites has become critical for understanding and mitigating mosquito-borne disease transmission in highly urbanized subtropical environments.

Previous studies have established that the probability of Aedes mosquito presence is directly linked to bite risk, with accurate quantification of mosquito abundance recognized as crucial for risk assessment [5,6]. Consequently, extensive research has examined how climatic, environmental, and socioeconomic factors influence *Aedes* distribution to evaluate bite risk [7,8]. However, assessing risk solely from the single dimension of *Aedes* mosquito abundance inadequately captures the complex relationship between *Aedes* bite risk and mosquito-borne disease transmission [9]. Few studies have attempted to integrate multiple risk dimensions, particularly those related to the likelihood of human-vector contact and population susceptibility. High human density inherently increases contact probability with Aedes mosquitoes [10]. Meanwhile, vulnerable populations, such as children, elderly individuals, low-income communities, and those with limited education [11,12], may face disproportionate risks of severe outcomes.

Traditional epidemiological models typically treated mosquito presence, population distribution, and human susceptibility as independent components rather than examining the spatial co-occurrence and interactions [13–15]. This fragmented approach limited the ability to identify where risks truly converge, where high mosquito density, high human exposure, and elevated vulnerability spatially overlap to create maximum transmission potential. Therefore, a comprehensive spatial assessment framework is needed that simultaneously quantifies all risk dimensions and, critically, identifies the spatial intersections to guide targeted public health interventions.

To address this gap, we adopted a risk assessment framework inspired by the risk framework proposed by Intergovernmental Panel on Climate Change (IPCC) [16], in which risk is conceptualized as a conbination of hazard, exposure, and vulnerability. This framework has been widely used to evaluate the risks associated with health-environment interactions [15,17]. We adapted this framework specifically for *Aedes* mosquito bite risk by: (i) defining hazard as spatial *Aedes* mosquito density; (ii) quantifying exposure through population distribution; (iii) assessing vulnerability via demographic and socioeconomic susceptibility factors. We hypothesized that the risk of *Aedes* mosquito-borne disease transmission is higher in locations where *Aedes* mosquito density and human exposure are simultaneously high, and that demographic and socioeconomic characteristics would further modify this risk.

To quantify these components, the Optimal Parameters-based Geographical Detector (OPGD) and Geographically Weighted Random Forest (GWRF) model were employed to estimate *Aedes* mosquito density as the hazard layer. High-resolution population distribution was adopted to represent exposure layer. Meanwhile, an Index of Multiple Deprivation (IMD), derived from Geographically Weighted Principal Component Analysis (GWPCA), was constructed to evaluate vulnerability. After conducting a spatial assessment of the hazard, exposure, and vulnerability, we generated a spatial distribution map of *Aedes* mosquito bite risk in Shenzhen. The framework was validated against dengue fever case data to confirm epidemiological relevance. Based on the identified hotspots and underlying factors contributing to *Aedes* mosquito bite risk, recommendations and measures to decrease these risks were proposed. The insights gained from this study may offer useful guidance for public health administrators in recognizing potentially high-risk areas and relevant contributing factors, and could support ongoing efforts to inform urban policies aimed at mitigating vector-borne disease risks.

## 2. Methods

### 2.1. Study area

**Fig 1:**
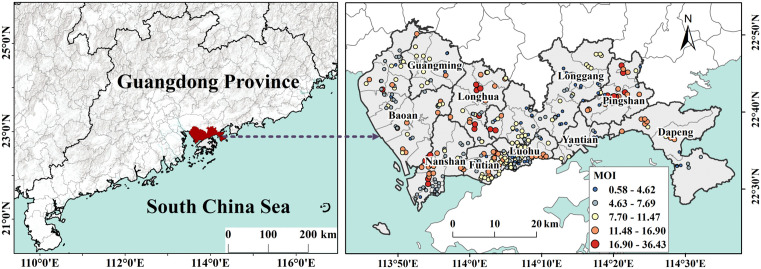
The study area and monitoring point locations in August 2022. Base map credit: This figure uses the standard map (Approval Number: GS(2023)2767) supervised by the Ministry of Natural Resources of the People’s Republic of China (http://bzdt.ch.mnr.gov.cn/). The boundary of the base map has not been modified.

The study was conducted in Shenzhen (22°27′-22°52′N, 113°46′-114°37′ E), a rapidly growing subtropical metropolitan area located in southern China within Guangdong Province, directly bordering Hong Kong (Fig 1). Covering an area of 1997.47 km², Shenzhen has a population exceeding 17 million, with a population density exceeding 8,000 people per square kilometer. Shenzhen’s subtropical climate, featuring mild winters and hot, humid summers, provides ideal conditions for the breeding and survival of *Aedes* mosquitoes.

This study primarily focused on the main urban area of Shenzhen, excluding the Shenzhen–Shanwei Special Cooperation Zone. The city comprises 10 administrative districts, including Guangming, Bao’an, Nanshan, Longhua, Futian, Luohu, Longgang, Yantian Pingshan, and Dapeng, which are further subdivided into 74 subdistricts. Each subdistrict serves as a fundamental urban administrative unit and constitutes the finest spatial unit available in census data. Therefore, the subdistrict level was employed as the smallest spatial analytical unit in this study.

### 2.2. Data source

#### 2.2.1. Ovitrap data.

Due to resource and logistical constraints, Shenzhen lacks large-scale human landing catch (HLC) or human double net (HDN) data for direct measurement of *Aedes* mosquito biting behavior. Shenzhen has established an *Aedes* mosquito surveillance network primarily based on the Breteau Index (BI) and the Mosquito Oviposition Index (MOI), focusing mainly on *Aedes albopictus*. The BI is mainly used for monitoring larval populations. Therefore, we quantified the adult *Aedes* mosquito density through the MOI, which was calculated as follows:


MOI=Number of positive ovitraps/Total number of ef fectively recovered ovitraps×100

The *Aedes* mosquito surveillance data was collected by Shenzhen Center for Disease Control and Prevention (Shenzhen CDC). The ovitrap method is recommended by the Chinese CDC and WHO [18] as a cost-effective and sensitive tool for *Aedes* mosquito surveillance, and has been widely adopted throughout China. A limited number of ovitraps is sufficient to determine vector presence. Moreover, previous studies have shown that fewer than 100 ovitraps can reliably estimate *Aedes* mosquito population density in large urban environment [19]. Given the high incidence of dengue and peak *Aedes* mosquito vector activity in summer, on-site data collection was carried out in August 2022 (Fig 1). A total of 415 monitoring points were included in the study. At each location, 60 mosquito ovitraps were installed. These monitoring points cover all subdistricts of Shenzhen. These traps remained in place for four days and were retrieved on the fifth day. Positive cases were recorded if adult mosquitoes or eggs were found in the ovitraps.

#### 2.2.2. Auxiliary variables.

According to previous studies, meteorological factors, social factors, urban landscape, and air pollution may influence the distribution and density of *Aedes* mosquitoes, and we collected 17 potential related factors. The data sources and the reasons for impacting *Aedes* mosquito density were presented in the S1 Table. The calculation of multicollinearity for auxiliary variables was performed using the Variance Inflation Factor (VIF), and variables with VIF > 10 were removed, leaving a total of 14 auxiliary variables. Climate factors encompass precipitation (PRE), relative humidity (RH), and temperature (TEM) [7,8,20,21]. Social factors encompass population density (POP), road networks density (Road), regional gross domestic product (GDP), land surface temperature (LST), and the intensity of night light (Night) [22–25]. Urban landscape encompasses distance to urban blue spaces (Distance), built areas density (Built), impervious surface area (GAIA), urban green surface density (Green), and normalized difference vegetation index (NDVI) [26–28]. Air pollution encompasses the ground-level PM10 [29].

#### 2.2.3. Demographic statistical data.

Demographic and socioeconomic data were obtained from the Seventh National Census and the 2022 Shenzhen Statistical Yearbook. This includes population structure data at the subdistrict level, categorized by age, education level, income, occupation, and housing conditions. These data were sourced from the official website of the Shenzhen Bureau of Statistics.

### 2.3. Framework

**Fig 2:**
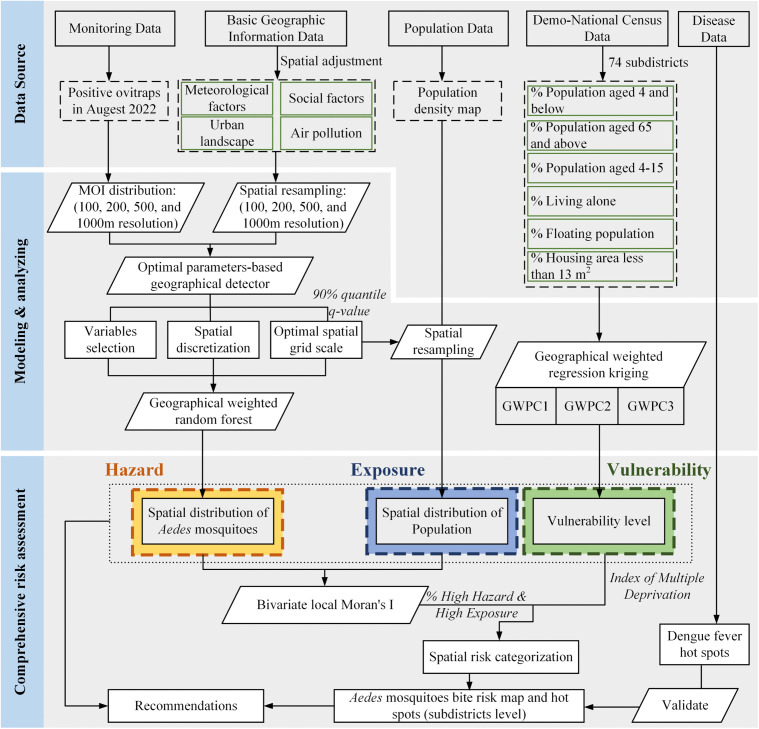
Framework of the *Aedes* mosquito bite risk mapping.

This framework primarily relies on cross-sectional data for risk assessment. Drawing on the risk framework, this study identified *Aedes* mosquito bite risk as a combination of hazard, exposure, and vulnerability layers. Accordingly, the *Aedes* mosquito bite risk map was generated as an integrated results of the *Aedes* mosquito hazard, human exposure, and population vulnerability layers. All layers were calculated at the subdistrict level, and the framework of this study was presented in Fig 2.

#### 2.3.1. Hazard and exposure layer.

First, we used OPGD model (Detailed descriptions of OPGD are provided in the S1 Method) to investigate the relationship between the spatial patterns of *Aedes* mosquitoes and various auxiliary variables. This analysis identified factors influencing *Aedes* mosquito density, assigning weights to each factor while excluding auxiliary variables with low *q*-values. Based on the selected factors and *Aedes* mosquito surveillance data, the GWRF model (Detailed descriptions of GWRF are provided in the S2 Method) was then applied to interpolate the spatial density of *Aedes* mosquito distribution in Shenzhen. To evaluate the performance of the GWRF model, its performance was compared against Geographically Weighted Regression (GWR) and Random Forest (RF), with detailed evaluation metrics provided in the S3 Method. The resulting *Aedes* mosquito distribution map served as the hazard layer.

Given that all residents face potential exposure to *Aedes* mosquitoes, population density was used as a surrogate indicator to quantify human exposure levels. The population density data in this study were derived from the research by Chen et al. (2024) [30], in which ensemble learning and big geospatial data to provide a more refined estimation method based on the Seventh National Census data.

We applied bivariate local Moran’s I [31] to detect clusters based on spatial autocorrelation between *Aedes* mosquito hazard and population exposure. This method has been used to examine population exposure to natural hazards [32]. High-risk areas were defined by high-high (HH) clusters of grids, where the proportion of HH grids to the total area was calculated for each subdistrict and used as a sub-index to integrate hazard and exposure.

#### 2.3.2. Vulnerability areas identification.

Socioeconomic deprivation encompasses various interrelated factors that affect living standards and the capacity of populations to cope with external health risk [33]. To assess such multidimensional disparities, the IMD has been widely employed to examine spatial patterns of vulnerability [34,35]. In the calculation of *Aedes* mosquito bite risk, vulnerability is shaped by the complex interplay of demographic, economic, and housing characteristics.

In this study, we applied GWPCA model to reduce the dimensionality of related demographic and socioeconomic dimensions and construct a localized IMD as a indicator of population vulnerability (Detailed descriptions of GWPCA and IMD calculation are provided in the S4 Method). Based on literature review and the national census data, we identified 12 indicator variables that may relate to *Aedes* mosquito bite vulnerability in Table 1. Using GWPCA, we extracted the principal components from these variables and constructed the IMD by assigning a unique deprivation score to each subdistrict. The resulting IMD map served as the vulnerability layer.

**Table 1:** Vulnerable groups for *Aedes* mosquito bites in Shenzhen.

Vulnerable groups	Reasons	Abbreviation	Indicator	References
Population aged 4 and below	Weaker immune system	Kid	Percentage of Kids aged 4 and below (%)	Dickin et al., 2013 [11]
Population aged 4–15	More likely to exposure to *Aedes* mosquitoes	Teenager	Percentage of teenagers aged 5–14 (%)	Dickin et al., 2013 [11]
Population aged 65 and above	More susceptible to viruses carried by *Aedes* mosquitoes	Elderly	Percentage of elderly aged 65 and above (%)	de Mattos Almeida et al., 2007 [36]
Population aged 15 and above with lower education levels	Weaker self-protection awareness	Low_edu	Percentage of people aged 15 and above with lower education (below middle school level) (%)	Dickin et al., 2013 [11]
Floating population	Higher probability of exposure to *Aedes* mosquito bites during commuting	Flow	Percentage of floating population with household registration outside the province (%)	Akter et al., 2017 [12]
Small housing area	Potentially lower socioeconomic status and poor living conditions	Small_hou	Percentage of population with per capita housing area less than 13 square meters (%)	Adnan et al., 2021 [37]
Engaged in primary industries (agriculture, forestry, animal husbandry, and fishing)	Greater exposure to high-risk environments	Agri_fish	Percentage of population engaged in primary industries (%)	Akter et al., 2017 [12]
Living alone	Less attention to self-protection	One_hou	Percentage of population living alone (%)	Adnan et al., 2021 [37]
High population density in residential area	Higher likelihood of exposure to *Aedes* mosquitoes	High_den	Population density (people per square kilometer)	de Mattos Almeida et al., 2007 [36]
Lower economic status	Weaker ability to protect oneself and seek medical care	Low_inc	Shenzhen GDP per capita/ District GDP per capita	Akter et al., 2017 [12]
Gender imbalance in the region	Males have more developed sweat glands, possibly attracting more *Aedes* mosquito bites	Gender	Sex ratio (female = 100)	Raude et al., 2012 [38]
High average household size	Potentially lower economic conditions	Lar_hou	Average number of people per household	de Mattos Almeida et al., 2007 [36]

#### 2.3.3. *Aedes* mosquito bite risk assessment map.

For a consistent analytical approach, the HH hazard-exposure score was also normalized using the min-max method, consistent with the procedure applied to the IMD, to enable integration into a composite risk index. Then we combined the HH hazard-exposure and vulnerability layers, generating a risk index and map at the subdistrict level. In this comprehensive risk assessment, equal weight was given to all three components. However, as hazard and exposure were combined into a single component, the HH hazard-exposure cluster contributed 2/3, while vulnerability contributed 1/3 [9]. Risk levels were categorized based on natural breaks, with subdistricts falling into the highest risk tier identified as hot spots. Subsequently, the key drivers behind each hot spot were investigated by evaluating hazard, exposure, and vulnerability outcomes [13,14].

## 3. Results

### 3.1. Spatial distribution of *Aedes* mosquitoes

#### 3.1.1. Optimal spatial grid scale and variable selections.

The effects of potential influencing factors on MOI were quantified across four different grid scales (100m × 100m, 200m × 200m, 500m × 500m, 1000m × 1000m), as presented in Table 2). The analysis revealed that the 90^th^ percentile of *q*-values reached its maximum (*q* = 0.51) at the 200 m × 200 m grid scale [39]. Consequently, 200 m was identified as the optimal scale for characterizing the spatial heterogeneity of MOI in Shenzhen.

**Table 2:** *Q*-values of potential influence factors at different spatial grid scales.

Auxiliary Variable	100m	200m	500m	1000m
LST	0.49	0.52	0.47	0.42
TEM	0.46	0.50	0.45	0.41
PRE	0.44	0.46	0.40	0.38
RH	0.42	0.46	0.40	0.39
PM10	0.40	0.41	0.35	0.32
Night	0.37	0.37	0.33	0.28
NDVI	0.30	0.32	0.27	0.23
Distance	0.29	0.32	0.27	0.22
POP	0.24	0.28	0.21	0.18
Road	0.22	0.26	0.20	0.14
Built	0.17	0.24	0.17	0.11
GDP	0.18	0.20	0.15	0.10
Green	0.07	0.07	0.05	0.04
GAIA	0.05	0.06	0.05	0.03
**90% quantile**	0.47	**0.51**	0.46	0.41

We employed five discretization methods (quantile, equal, natural, standard deviation, and geometric) with five interval types (3–7 categories) to calculate the *q*-values of 14 influencing factors. The optimal method was selected based on maximum *q*-value for each factor (S1 Fig). Table 3 showed the Pearson correlation coefficients and *q*-values between MOI and influencing factors. Correlation analysis showed that MOI was significantly correlated with most factors, except Green (p = 0.65). LST (r = 0.71, p < 0.01) had the strongest correlation with MOI, while GAIA (r = 0.04, p = 0.07) exhibited the weakest correlation with MOI. PM10 (r = -0.59, p < 0.01), Night (r = -0.44, p = 0.02), Road (r = -0.28, p = 0.02), and GDP (r = -0.16, p = 0.06) showed a negative correlation with MOI. LST had the highest *q*-value (q = 0.52), followed by TEM (q = 0.50), PRE (q = 0.46), RH (q = 0.46), PM_10_ (q = 0.41), Night (q = 0.37). The results of *q*-values indicated that LST, TEM, PRE, RH, PM10 and Night explained 52%, 50%, 46%, 46%, 41%, and 37% of the spatial variation of MOI, respectively. These findings suggested that OPGD was a viable model for investigating the spatial heterogeneity mechanisms of MOI. According to the results of *q*-values, we excluded Green and GAIA with *q*-value < 0.15, and considered the remaining variables to have a significant impact on the spatial heterogeneity of MOI.

**Table 3:** Pearson correlation coefficients and *q*-values between MOI and influencing factors.

Auxiliary Variable	r	*q*-value
LST	0.71***	0.52***
TEM	0.62***	0.50***
PRE	0.64***	0.46***
RH	0.58***	0.46***
PM10	-0.59***	0.41***
Night	-0.44**	0.37***
NDVI	0.39**	0.32**
Distance	0.32***	0.32**
POP	0.35*	0.28***
Road	-0.28**	0.26**
Built	0.17*	0.24**
GDP	-0.16*	0.20*
Green	-0.11	0.07
GAIA	0.04*	0.06*

*Note:* r is Pearson’s correlation coefficient. *, **, and *** indicate the significant level of 0.1, 0.05, and 0.01, respectively.

#### 3.1.2. Prediction performance and mosquito density distribution.

Table 4 presented the accuracy metrics of prediction models. The GWRF outperformed GWR and RF models, yielding the lowest MAE (2.04), RMSE (2.44), and MAPE (43.45%), as well as the highest R^2^ (0.82).

**Table 4:** Evaluation results of different spatial prediction models.

Model	MAE	RMSE	R^2^	MAPE
GWR	3.97	4.35	0.68	72.43%
RF	3.06	3.67	0.71	58.84%
GWRF	2.04	2.44	0.82	43.45%

**Fig 3:**
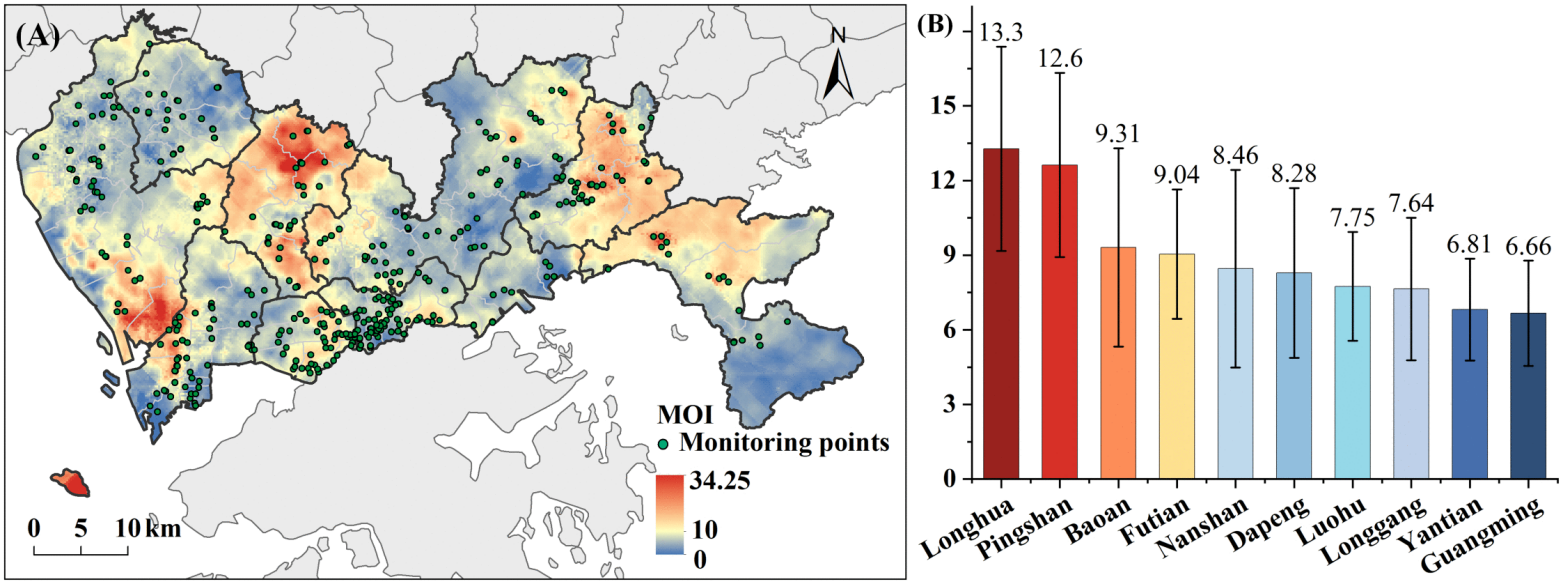
The spatial distribution of MOI. (A) The MOI map. (B) The average MOI in each district. Base map credit: This figure uses the standard map (Approval Number: GS(2023)2767) supervised by the Ministry of Natural Resources of the People’s Republic of China (http://bzdt.ch.mnr.gov.cn/). The boundary of the base map has not been modified.

Combining the MOI-related auxiliary variable in Shenzhen citywide, we predicted the spatial distribution of MOI at 200m spatial resolution (Fig 3A). The MOI was relatively high in the southern Bao’an, northern Longhua, and the central Pingshan, while it was comparatively low in Guangming and Yantian. Fig 3B displayed the average MOI across districts, Longhua exhibited the highest average MOI at 13.27, followed by Pingshan with an average of 12.62. In contrast, Guangming had the lowest average MOI at 6.66.

### 3.2. Spatial pattern of *Aedes* mosquito bite risk

#### 3.2.1. Descriptive statistic of the vulnerable groups.

The descriptive statistics of vulnerable groups across subdistricts are summarized in Table 5, with spatial distributions detailed in S2 Table and S2 Fig. Our analysis reveals distinct spatial clustering of socioeconomic and demographic vulnerabilities. The central districts of Longgang and Luohu exhibited higher concentrations of young populations (children and teenagers). In contrast, the more established urban cores of Nanshan, Futian, Luohu, and Yantian were characterized by older populations, larger household sizes, and higher income levels. Notably, the northwestern and northeastern subdistricts, which showed higher proportions of floating populations and single-person households, concurrently displayed smaller per capita living spaces. Additionally, the highest population densities were clustered in southern Longhua and the western parts of Luohu and Futian. These patterns highlight a clear spatial segregation of vulnerability factors within Shenzhen.

**Table 5:** Descriptive statistics of the subdistricts.

	Min.	1st Qu.	Median	3rd Qu.	Max.	Mean
Kid	3.314	4.992	5.556	5.945	7.429	5.527
Teenager	5.826	8.723	9.832	10.793	12.941	9.741
Elderly	1.119	2.058	3.361	5.094	7.928	3.774
Low_edu	3.791	5.566	7.260	8.671	20.296	7.626
Flow	19.40	30.47	41.41	57.61	74.60	44.05
Small_hou	5.393	19.565	27.610	34.528	53.388	26.764
Agri_fish	0.020	0.081	0.112	0.124	0.936	0.162
One_hou	19.20	29.50	38.73	44.67	52.52	37.27
High_den	145.5	6435.3	11126.6	20083.2	56844.4	14526.4
Low_inc	8.594	10.292	14.613	30.371	36.348	19.163
Gender	94.12	108.72	121.08	134.21	175.28	123.25
Lar_hou	1.800	2.080	2.350	2.627	3.090	2.355

#### 3.2.2. Geographically weighted principal component analysis and spatial vulnerability pattern.

**Fig 4:**
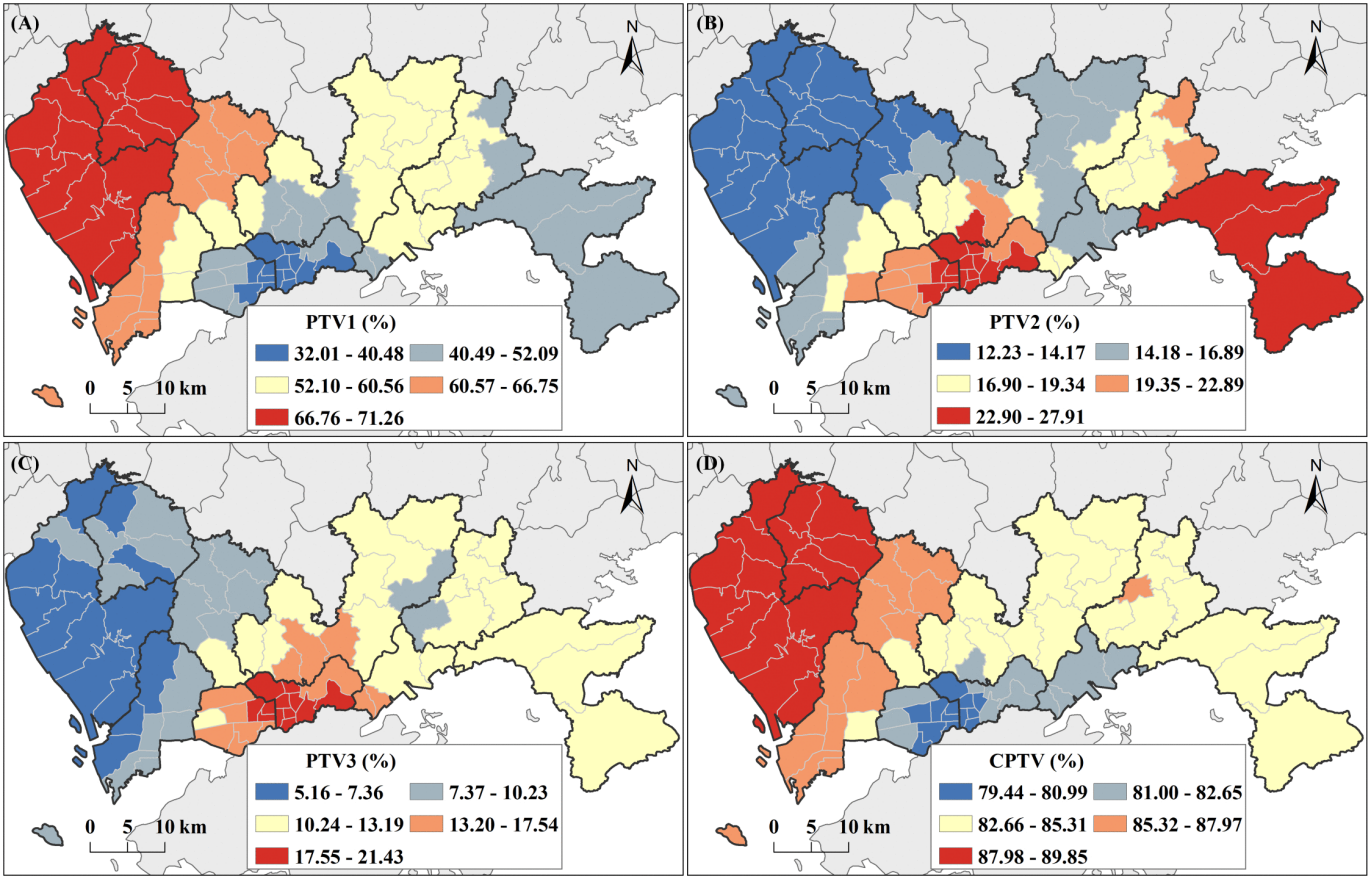
Spatial distribution of PTV generated by GWPCA. (A)–(C) PTV for GWPC1–GWPC3. (D) CPTV for the sum of these GWPC. Base map credit: This figure uses the standard map (Approval Number: GS(2023)2767) supervised by the Ministry of Natural Resources of the People’s Republic of China (http://bzdt.ch.mnr.gov.cn/). The boundary of the base map has not been modified.

**Fig 5:**
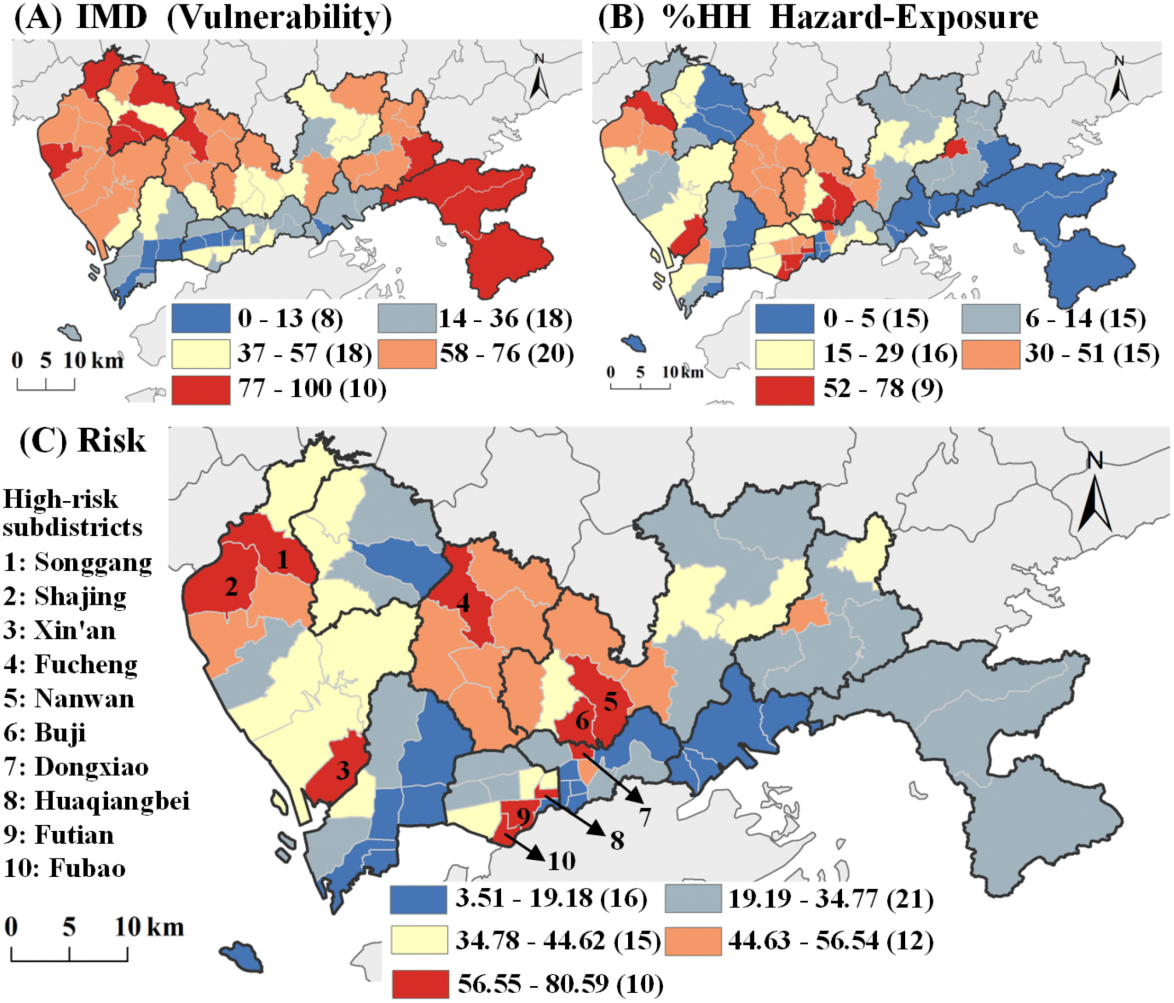
Maps for *Aedes* mosquito bite risk assessment in Shenzhen. A) Spatial pattern of vulnerability; B) Local spatial association of HH hazard-exposure; C) *Aedes* mosquito bite risk assessment map. Base map credit: This figure uses the standard map (Approval Number: GS(2023)2767) supervised by the Ministry of Natural Resources of the People’s Republic of China (http://bzdt.ch.mnr.gov.cn/). The boundary of the base map has not been modified.

Fig 4 depicted the local proportion of total variation (PTV) and cumulative PTV (CPTV) for the selected first three GWPCs in Shenzhen. The GWPCA results showed that GWPC1 contributed most of the PTV in Baoan and Guangming, GWPC2 contributed most of the PTV in Luohu, Futian, and Dapeng, and GWPC3 contributed most of the PTV in Luohu and Futian. In all subdistricts in Shenzhen, these three GWPCs totally explained over 79.44% of the total variance, and peaked in the western subdistricts. According to the rotated geographical component matrixes, the highest local loadings (i.e., the winning variable) of GWPCs exhibited distinct spatial variation and a clear clustering pattern across different subdistricts. In northwestern Shenzhen, Small_hou had the highest local loadings for GWPC1, while in the central region, Elderly dominated. In Luohu, Yantian, and western Longgang, One_hou emerged as the top contributor to GWPC1, whereas in Dapeng and Pingshan, Gender was most influential. For GWPC2 and GWPC3, Kid and High_den generally exhibited the highest local loadings across most areas of Shenzhen. However, some subdistricts had other factors as winning variables, reflecting localized variations in the dominant contributors. S3 Fig. illustrated the spatially distribution of winning variables for the three GWPCs. Fig 5A showed the spatial distribution of integrated vulnerability, with subdistricts exhibiting high vulnerability primarily concentrated in Shenzhen’s northwest and southeast regions.

#### 3.2.3. High hazard and high exposure areas.

The exposure layer (S4 Fig) revealed substantial spatial heterogeneity in population distribution across Shenzhen. High population densities were concentrated in the central urban core, particularly in southern Longhua, western Luohu, and Futian districts. In contrast, the peripheral areas in Guangming (northwest), Dapeng (east), Pingshan (northeast), and Yantian (southeast) exhibited considerably lower population densities. By combining the hazard layer (Fig 3A), we applied the bivariate local Moran’s I to identify spatial clusters that reflect the spatial autocorrelation between *Aedes* mosquito hazard and population exposure. As shown in the LISA cluster map (Fig 5B), 18.54% of all grids were classified as HH clusters. Among the 74 subdistricts, five had no HH grids, whereas 11 subdistricts had more than 50% of area covered by HH grids. Longhua exhibited the highest proportion of HH grids, with an average coverage of 35.58% across its subdistricts in Futian. In contrast, subdistricts in Dapeng showed the lowest HH grid coverage, averaging only 1.04%. After min–max normalization, the spatial distribution of the HH exposure–hazard levels across subdistricts was illustrated in S5 Fig.

#### 3.2.4. *Aedes* mosquito bite risk map.

Fig 5C presented the spatial distribution of *Aedes* mosquito bite risk across Shenzhen. Validation using dengue fever case data showed that that the *Aedes* mosquito bite risk score were significantly associated with reported cases (August 2022: r = 0.446, p < 0.05), with similar correlations observed in the following months (S3 Table and S6 and S7 Figs).

A total of 10 subdistricts were in the highest risk category with a risk index exceeding 56.55, while 12 subdistricts were classified as moderate-high risk with indices ranging from 44.63 to 56.54. Among the 10 subdistricts with the highest *Aedes* mosquito bite risk, eight (Songgang, Xin’an, Nanwan, Buji, Dongxiao, Huaqiangbei, Futian, and Fubao) exhibited high hazard–exposure levels, with HH proportions exceeding 52%, placing them in the highest hazard-exposure category. In the other two subdistricts, Fucheng recorded a notably high IMD score (80.43). Shajing, with an HH proportion nearing the high-risk threshold at 51.19%, also registered an elevated IMD score of 69.59. The results indicated that *Aedes* mosquito bite risk exhibited significant clustering, with higher risk observed in Longhua, the southwestern part of Longgang, the northwestern part of Bao’an, and the southern part of Futian, while lower risk was identified in Nanshan and Yantian. The hot spots in southern Futian were mainly resulted from HH hazard-exposure. Although Guangming, Pingshan, and Dapeng had high IMD scores, the low HH hazard-exposure levels resulted in a reduced *Aedes* mosquito bite risk.

## 4. Discussion

This study developed a comprehensive spatial assessment framework that integrates hazard, exposure, and vulnerability dimensions to evaluate *Aedes* mosquito bite risk in Shenzhen. Unlike traditional approaches that focus solely on vector distribution, our framework provided a multidimensional perspective by simultaneously considering *Aedes* mosquito density, population distribution, and socioeconomic vulnerability. This integrated approach addresses a critical gap in vector-borne disease risk assessment, as previous studies have typically examined these components independently rather than analyzing spatial convergence. The application of advanced spatial modeling techniques, including OPGD for factor selection, GWRF for MOI prediction, and GWPCA for vulnerability assessment, enabled us to capture the spatial heterogeneity of *Aedes* mosquito bite risk.

### 4.1. Spatial patterns of hazard, exposure, and vulnerability

The optimal spatial scale of 200m × 200m identified through OPGD reflected the fine-grained environmental variations that influence *Aedes* mosquito ecology in urban settings. At this optimal grid resolution, the spatial relationships between the MOI and its influencing factors can be more clearly observed, facilitating a comprehensive exploration of the spatial heterogeneity of *Aedes* mosquito distribution across Shenzhen [40,41]. Natural environmental factors exerted a stronger influence on the spatial heterogeneity of *Aedes* mosquito distribution than socioeconomic conditions, which is consistent with previous findings [42,43]. The strong effects of LST (*q* = 0.52), TEM (*q* = 0.50), PRE (*q* = 0.46), and RH (*q* = 0.46) align with existing evidence that thermal and moisture conditions play a crucial role in regulating *Aedes* mosquito breeding and survival [44–47].

The GWRF model demonstrated superior performance (R^2^ = 0.82) compared with traditional GWR and RF models, underscoring the importance of considering both spatial non-stationarity and nonlinearity when predicting *Aedes* mosquito distribution in complex metropolitan areas. Our analysis revealed pronounced spatial clustering of *Aedes* mosquito, with elevated levels concentrated in southern Bao’an, northern Longhua, and central Pingshan.

The pronounced spatial concentration of population in Shenzhen’s central districts amplifies exposure risk in these areas. High-density residential zones in southern Longhua and the eastern Futian create conditions where large populations are potentially exposed to *Aedes* mosquitoes. The LISA cluster map (Fig 5B) identified substantial overlap between high mosquito density and high population exposure. These convergence zones represent areas where transmission risk is amplified by the simultaneous presence of vectors and human hosts [48].

The GWPCA-derived vulnerability assessment revealed distinct spatial patterns of socioeconomic deprivation across Shenzhen, with significantly lower deprivation levels observed in Nanshan, Futian, Luohu, and Yantian districts. These districts, historically comprising Shenzhen’s original Special Economic Zone core established in 1980s [49], demonstrated superior socioeconomic conditions consistent with early development advantage.

### 4.2. High-risk hotspots and intervention strategies

The integrated risk map identified 10 subdistricts with the highest *Aedes* mosquito bite risk, predominantly located in Longhua, southwestern Longgang, northwestern Bao’an, and southern Futian. Importantly, these hotspots exhibited heterogeneous risk profiles driven by different combinations of hazard, exposure, and vulnerability factors. For hotspots characterized by HH hazard–exposure overlaps (e.g., Songgang, Xin’an, Nanwan, Buji, Dongxiao, Huaqiangbei, Futian, and Fubao), intervention strategies should prioritize environmental management and vector control measures. These include enhanced surveillance, source reduction campaigns in residential and commercial areas, and focused larviciding programs in high-density residential zones. Practical measures such as improving drainage systems and maintaining well-managed green spaces can effectively reduce *Aedes* mosquito breeding habitats.

In contrast, hotspots driven by elevated vulnerability despite moderate hazard-exposure levels (Fucheng and Shajing) require socially targeted interventions. These include tailored health education programs for communities with large migrant populations and low educational attainment, the distribution of personal protective supplies (e.g., repellents and mosquito nets) to vulnerable households, and improvements to housing conditions and environmental sanitation in economically disadvantaged areas.

### 4.3. Validation and epidemiological relevance

The composite risk score demonstrated the strongest and most consistent correlation with dengue cases across all three months (Augest: r = 0.446, September: r = 0.397, in, October: r = 0.406; all *p* < 0.05), confirming that the integrated framework successfully captures disease transmission patterns. Within this framework, the HH hazard-exposure score showed consistently significant correlations with dengue cases (Augest: r = 0.412, September: r = 0.388, in, October: r = 0.402; all *p* < 0.05), indicating that areas with overlapping high mosquito density and dense human populations are indeed at elevated risk for disease transmission [50,51]. In contrast, MOI showed weak or non-significant correlations in September and October, suggesting that *Aedes* mosquito presence without considering human exposure provides limited predictive value [52]. The IMD (vulnerability) component was not independently correlated with dengue incidence, suggesting that vulnerability alone does not directly determine transmission dynamics.

These validation results confirm the superiority of integrated risk assessment over single-component approaches, with the composite risk score consistently outperforming individual components in predicting disease occurrence. The framework’s temporal stability across multiple months suggests it can reliably guide intervention planning throughout the transmission season, enabling public health authorities to prioritize resources toward subdistricts where transmission is most likely to occur.

### 4.4. Implementation of policies

The high-resolution map of *Aedes* mosquitoes (Fig 3) developed in this study serves as a valuable tool for urban managers to identify optimal locations for mosquito trap placement. The spatial patterns of *Aedes* mosquito bite risk in Shenzhen (Fig 5) revealed distinct hotspots within high-risk zones, providing critical insights for local governments and relevant authorities to understand the underlying risk factors and implement targeted interventions. These findings offer actionable knowledge for optimizing resource allocation and enhancing the effectiveness of *Aedes* mosquito control strategies across the city. Additionally, this study can be served as a model for *Aedes* mosquito control efforts in other high-density subtropical cities.

Our findings have important implications for sustainable urban development and vector-borne disease prevention in rapidly growing subtropical cities. The spatial heterogeneity of the vulnerability layer highlights the need to prioritize vulnerable populations in urbanization policies. Rapidly developing areas require proactive planning to prevent the emergence of high-risk zones.

### 4.5. Limitations and future works

Due to the lack of direct human biting rate data, the MOI was used as a proxy indicator. In addition, species-level differentiation of *Aedes* mosquitoes was not possible because of data limitations, despite behavioral differences among species. Demographic and socioeconomic data were also limited to the subdistrict scale, constraining the precision of vulnerability assessments, particularly given the large variations in subdistrict area between urban and suburban regions.

Future studies should collect higher-resolution data on *Aedes* mosquito abundance, species composition, and local demographic characteristics to refine analyses of vulnerability and exposure. Considering the combined effects of climate change, population aging, and socioeconomic disparities, the spatial patterns of *Aedes* mosquito bite risk in Shenzhen are expected to evolve over time. Subsequent research will therefore focus on exploring spatiotemporal variations in *Aedes* mosquito-related risks under different environmental and social scenarios.

## 5. Conclusion

The proposed framework and assessment model provided a robust approach to understanding and addressing the factors driving high-risk areas for *Aedes* mosquito bites. This study underscored the importance of integrating spatial analysis with public health strategies, enabling urban planning and health authorities to implement precise, location-specific measures to reduce the risk of *Aedes* mosquito bites. Moreover, the framework offers a reference for assessing vector-borne bite risks or infectious disease transmission risks in other high-density cities, particularly under the challenges of global climate change. Such efforts can help address future challenges, improve public health outcomes, and mitigate negative impacts on population health.

## Supporting information

S1 Method Optimal parameters-based geographical detector (OPGD). (DOC)

S2 Method Geographical weighted random forest (GWRF). (DOC)

S3 Method Evaluation and validation indicators. (DOC)

S4 Method Geographical weighted principal component analysis (GWPCA) and Index of Multiple Deprivation (IMD) calculation. (DOC)

S1 Table Potential Variables Influencing MOI. (DOC)

S2 Table Specific spatial distribution of different population characteristics. (DOC)

S3 Table Pearson correlation coefficients between the number of dengue fever cases and risk assessment components. (DOC)

S1 Fig Process and result of optimal spatial discretization for the Aedes mosquito density influencing factors. (TIF)

S2 Fig The specific spatial distribution of different population characteristics. Base map credit: This figure uses the standard map (Approval Number: GS(2023)2767) supervised by the Ministry of Natural Resources of the People’s Republic of China (http://bzdt.ch.mnr.gov.cn/). The boundary of the base map has not been modified.
(TIF)

S3 Fig Winning variables for the first three GWPCs in Shenzhen. (A) GWPC1; (B) GWPC2; (C) GWPC3. Base map credit: This figure uses the standard map (Approval Number: GS(2023)2767) supervised by the Ministry of Natural Resources of the People’s Republic of China (http://bzdt.ch.mnr.gov.cn/). The boundary of the base map has not been modified.
(TIF)

S4 Fig Exposure layer: Population distribution map (200m spatial resolution). Base map credit: This figure uses the standard map (Approval Number: GS(2023)2767) supervised by the Ministry of Natural Resources of the People’s Republic of China (http://bzdt.ch.mnr.gov.cn/). The boundary of the base map has not been modified.
(TIF)

S5 Fig High hazard and high exposure grids proportion map (after normalization). Base map credit: This figure uses the standard map (Approval Number: GS(2023)2767) supervised by the Ministry of Natural Resources of the People’s Republic of China (http://bzdt.ch.mnr.gov.cn/). The boundary of the base map has not been modified.
(TIF)

S6 Fig The spatial distribution of MOI. (A) The MOI map in September, 2022; (B) The MOI map in October, 2022 (200m spatial resolution). Base map credit: This figure uses the standard map (Approval Number: GS(2023)2767) supervised by the Ministry of Natural Resources of the People’s Republic of China (http://bzdt.ch.mnr.gov.cn/). The boundary of the base map has not been modified.
(TIF)

S7 Fig Maps for Aedes mosquito bite risk assessment in Shenzhen, September and October, 2022. (A) Spatial pattern of vulnerability; (B) Local spatial association of HH hazard-exposure in September; (C) Local spatial association of HH hazard-exposure in October; (D) *Aedes* mosquito bite risk assessment map in September; (E) *Aedes* mosquito bite risk assessment map in October. Base map credit: This figure uses the standard map (Approval Number: GS(2023)2767) supervised by the Ministry of Natural Resources of the People’s Republic of China (http://bzdt.ch.mnr.gov.cn/). The boundary of the base map has not been modified.
(TIF)

S1 Data Original data of MOI and social and economic factors. (DOCX)
