# Supplementary material for: Spatial pattern assessment of Aedes mosquito bite risk in a subtropical metropolitan area: A case study in Shenzhen
Source: PLoS Negl Trop Dis. 2025 Dec 23;19(12):e0013843. doi: 10.1371/journal.pntd.0013843 (PMC12725540; doi:10.1371/journal.pntd.0013843)
Supplement: S1 Method — (DOC) [file pntd.0013843.s001.doc]

**S1_Method.** Optimal parameters-based geographical detector (OPGD)

The OPGD model [1] is an optimized method based on the geographical detector (GD) [2]. It improves model performance and result accuracy by adjusting parameters such as thresholds and weights, effectively addressing challenges related to the spatial discretization of continuous variables and the selection of spatial scales. In this study, the OPGD model was used to identify the optimal spatial parameters for detecting the spatial relationships between potential influencing factors and the distribution of *Aedes* mosquitoes, including the optimal selection of spatial discretization methods and breakpoint numbers. The OPGD model typically uses *q*-value to reveal the relative importance of explanatory variables. The *q*-value is determined by comparing the variance of observations across the study area with the variance within individual variable layers. The *q*-value is calculated using the following formula:

$$q=1-\frac{\sum_{h=1}^{L} N_{h}\sigma_{h}^{2}}{N\sigma^{2}}=1-\frac{SSW}{SST}$$

Where $h=1,\text{…,}L$ represents the stratification of potential influencing factors; $N$and$N_{h}$ represent the number of spatial grid in Shenzhen and strata $h$, respectively; $\sigma^{2}$ and $\sigma_{h}^{2}$ represent the variance of *Aedes* mosquito density in Shenzhen and within strata $h$, respectively;$SST=N\sigma^{2}$represents the total variance in Shenzhen; and $SSW=\sum_{h=1}^{L} N_{h}\sigma_{h}^{2}$ represents the sum of within-strata variance. *q*-value ranges from 0 to 1, where a higher *q*-value indicates a stronger explanatory power of the influencing factors on the *Aedes* mosquito density.

**References:**

1. Song Y, Wang J, Ge Y, Xu C. An optimal parameters-based geographical detector model enhances geographic characteristics of explanatory variables for spatial heterogeneity analysis: cases with different types of spatial data. GIScience Remote Sens. 2020 [cited 13 Jan 2025]. Available: https://www.tandfonline.com/doi/abs/10.1080/15481603.2020.1760434

2. Wang J-F, Zhang T-L, Fu B-J. A measure of spatial stratified heterogeneity. Ecol Indic. 2016;67: 250–256. doi:10.1016/j.ecolind.2016.02.052
