# Supplementary material for: Spatial pattern assessment of Aedes mosquito bite risk in a subtropical metropolitan area: A case study in Shenzhen
Source: PLoS Negl Trop Dis. 2025 Dec 23;19(12):e0013843. doi: 10.1371/journal.pntd.0013843 (PMC12725540; doi:10.1371/journal.pntd.0013843)
Supplement: S2 Method — (DOC) [file pntd.0013843.s002.doc]

**S2_Method.** Geographical weighted random forest (GWRF)

Due to the presence of spatial non-stationarity [1,2], the relationships between variables often change with geographic location. In 1996, Fotheringham proposed the Geographically Weighted Regression (GWR) model [3], which applies regression analysis to individual points within the study area. A local regression model is established for each sample point, and the model is then solved to derive regression coefficients at different spatial locations. These coefficients quantitatively reflect the spatial heterogeneity of variable relationships due to changes in spatial position. The formula for the GWR model is as follows:

$$y_{i}=\beta_{0}(m_{i},n_{i})+\sum_{j=1}^{k} \beta_{j}(m_{i},n_{i})x_{ij}+\varepsilon_{i}$$

In this study, $y_{i}(m_{i},n_{i})$ is the value of *Aedes* mosquito density at location $(m_{i},n_{i})$; $x_{ij}$is the value of auxiliary variable $(m_{i},n_{i})$; $\beta_{0}(m_{i},n_{i})$ is the intercept; $\beta_{j}(m_{i},n_{i})$ is the regression coefficient; $k$ represents the total number of auxiliary variables; $\varepsilon$represents the residual.

However, since the relationships between predictors and *Aede*s mosquito distribution may also be nonlinear, this study further integrated random forest (RF) with the GWR framework to develop a Geographically Weighted Random Forest (GWRF) model. The GWRF hybrid approach captures both nonlinear associations and spatial dependencies. It constructs an independent random forest model at each spatial location, incorporating geographical weighting of samples to enhance local prediction accuracy.

The formula for the GWRF model is as follows:

$$y_{i}=a(m_{i},n_{i})x_{i}+\varepsilon,i=1,2,3,\text{…,}n$$

Where $a(m_{i},n_{i})x_{i}$ is the locally calibrated RF non-linear prediction function at location $(m_{i},n_{i})$, $\varepsilon$ represents the residual. In the implementation of the GWRF, two key parameters governing model performance are the number of decision trees (ntree) and the number of candidate variables for splitting at each tree node (mtry). We primarily followed the recommended values from the common practices in the field, setting them to 500 and 7, respectively [4].

**References:**

1. Bhunia GS, Shit PK, Pourghasemi HR, Edalat M. 19 - Prediction of Soil Organic Carbon and its Mapping Using Regression Analyses and Remote Sensing Data in GIS and R. In: Pourghasemi HR, Gokceoglu C, editors. Spatial Modeling in GIS and R for Earth and Environmental Sciences. Elsevier; 2019. pp. 429–450. doi:10.1016/B978-0-12-815226-3.00019-3

2. Wang D, Li X, Zou D, Wu T, Xu H, Hu G, et al. Modeling soil organic carbon spatial distribution for a complex terrain based on geographically weighted regression in the eastern Qinghai-Tibetan Plateau. CATENA. 2020;187: 104399. doi:10.1016/j.catena.2019.104399

3. Brunsdon C, Fotheringham AS, Charlton ME. Geographically Weighted Regression: A Method for Exploring Spatial Nonstationarity. 1996 [cited 13 Jan 2025]. Available: https://onlinelibrary.wiley.com/doi/10.1111/j.1538-4632.1996.tb00936.x

4. Belgiu M, Drăguţ L. Random forest in remote sensing: A review of applications and future directions. ISPRS J Photogramm Remote Sens. 2016;114: 24–31. doi:10.1016/j.isprsjprs.2016.01.011
