# Supplementary material for: Spatial pattern assessment of Aedes mosquito bite risk in a subtropical metropolitan area: A case study in Shenzhen
Source: PLoS Negl Trop Dis. 2025 Dec 23;19(12):e0013843. doi: 10.1371/journal.pntd.0013843 (PMC12725540; doi:10.1371/journal.pntd.0013843)
Supplement: S3 Method — (DOC) [file pntd.0013843.s003.doc]

**S3_Method.** Evaluation and validation indicators

To evaluate the performance of the GWRF model, we compared it against both GWR and RF. The *in-situ* monitoring dataset was randomly split into training (70%) and validation (30%) subsets. Spatial predictions were generated on a 200m × 200m grid. Model performance was assessed using the mean absolute error (MAE), root mean square error (RMSE), mean absolute percentage error (MAPE), and the determination coefficient (R^2^).

$$MAE=\frac{1}{n}\sum_{i=1}^{n} \left|y_{i}-\hat{y}_{i}\right|$$

$$RMSE=\sqrt{\frac{1}{n}\sum_{i=1}^{n} (y_{i}-\hat{y}_{i})^{2}}$$

$$R^{2}=1-\frac{\sum_{i=1}^{n} (y_{i}-\hat{y}_{i})^{2}}{\sum_{i=1}^{n} (y_{i}-\bar{y}_{i})^{2}}$$

$$MAPE=\frac{1}{n}\sum_{i=1}^{n} \left|\frac{y_{i}-\hat{y}_{i}}{y_{i}}\right|\times100\%$$

where $y_{i}$ and $\hat{y}_{i}$ denoted the observed and predicted MOI at the location $(m_{i},n_{i})$, $\bar{y}_{i}$ presented the average value of the MOI, and $n$ was the sample size.
