# Supplementary material for: Spatial pattern assessment of Aedes mosquito bite risk in a subtropical metropolitan area: A case study in Shenzhen
Source: PLoS Negl Trop Dis. 2025 Dec 23;19(12):e0013843. doi: 10.1371/journal.pntd.0013843 (PMC12725540; doi:10.1371/journal.pntd.0013843)
Supplement: S4 Method — (DOC) [file pntd.0013843.s004.doc]

**S4_Method.** Geographical weighted principal component analysis (GWPCA) and Index of Multiple Deprivation (IMD) calculation

Extending traditional principal component analysis (PCA) to geographic space is highly significant for addressing spatial heterogeneity in multivariate data [1,2]. By incorporating a spatial weight matrix and geographic locations into PCA calculations, the GWPCA model is constructed. In this study, GWPCA was applied to develop the Index of Multiple Deprivation (IMD), as the local principal components and variance derived from GWPCA provide valuable insights for constructing the IMD [3]. Assuming the spatial coordinates $(u,v)$ of a spatial variable $X$, the geographically weighted variance-covariance matrix $\sum(u,v)$, which is associated with the geographic coordinates in GWPCA, is formulated as follows:

$$\sum(u,v)=X^{\top}W(u,v)X$$

Where $X$ is the data matrix of vulnerability-influencing factors. The calculation formulas for local eigenvalues and eigenvectors are as follows:

$$L(u,v)V(u,v)L(u,v)^{\top}=\sum(u,v)$$

Where $L(u,v)$ is the data matrix of local eigenvectors, $V(u,v)$ is the data matrix of local eigenvalues. To reduce noise impact and identify key vulnerability factors, this study retains the first 3 PCs with eigenvalues [4]. GWPCA calculates the weights for each dimension by multiplying the squared local loadings by the variance explained by each corresponding principal component and summing across components. The formula is as follows:

$$W_{j}=\sum_{j=1,2,3} PC_{j,i}^{2}\times\frac{\sqrt{\lambda_{j}}}{\sum_{j=1,2,3} \sqrt{\lambda_{j}}}$$

Where $W_{j}$is the weight of *j^th^* GWPC，$PC_{j,i}$is the local loadings of *j^th^* GWPC，$\lambda_{j}$is the eigenvalue of *j^th^* GWPC. The initial vulnerability index at each street level is set as $S_{n}$:

$$S_{n}=\sum_{j=1}^{m} C_{n,j}W_{j}$$

Where $C_{n,j}$ is the local value for *j^th^* GWPC of street n, $W_{j}$ is the combined weight of IMD components for *k^th^* GWPCs for street n, m=3.

The IMD for each street in Shenzhen was standardized using the min-max normalization method, as shown in the following formula:

$$IMD=\frac{S_{n}-S_{\min}}{S_{\max}-S_{\min}}\times100$$

Where $S_{n}$, $S_{\max}$, $S_{\min}$is the initial deprivation score for street n, the highest and lowest values of the initial deprivation score. An IMD value of “0” indicates the street with the lowest ranking, while the highest-ranking street corresponds to an IMD value of “100”. The IMD values of other streets vary between “0” and “100”. A higher IMD value corresponds to a greater vulnerability of *Aedes* mosquito bites.

**References:**

1. Fernández S, Cotos-Yáñez T, Roca-Pardiñas J, Ordóñez C. Geographically Weighted Principal Components Analysis to assess diffuse pollution sources of soil heavy metal: Application to rough mountain areas in Northwest Spain. Geoderma. 2018;311: 120–129. doi:10.1016/j.geoderma.2016.10.012

2. Harris P, Brunsdon C, Charlton M. Geographically weighted principal components analysis. Int J Geogr Inf Sci. 2011;25: 1717–1736. doi:10.1080/13658816.2011.554838

3. Mishra SV. Urban deprivation in a global south city-a neighborhood scale study of Kolkata, India. Habitat Int. 2018;80: 1–10. doi:10.1016/j.habitatint.2018.08.006

4. Das A, Ghosh S, Das K, Basu T, Dutta I, Das M. Living environment matters: Unravelling the spatial clustering of COVID-19 hotspots in Kolkata megacity, India. Sustain Cities Soc. 2021;65: 102577. doi:10.1016/j.scs.2020.102577
