# Supplementary material for: Spatial pattern assessment of Aedes mosquito bite risk in a subtropical metropolitan area: A case study in Shenzhen
Source: PLoS Negl Trop Dis. 2025 Dec 23;19(12):e0013843. doi: 10.1371/journal.pntd.0013843 (PMC12725540; doi:10.1371/journal.pntd.0013843)
Supplement: S1 Table — (DOC) [file pntd.0013843.s005.doc]

According to previous studies, this study selected auxiliary variables for the following four categories: Meteorological factors, social factors, urban landscape, and air pollution. Detailed description and sources of auxiliary variables were shown in S1_Table.

**S1_Table. Potential Variables Influencing MOI**

| **Auxiliary variables** | **Types** | **Spatial resolution** | **Time period** | **Data source** |
| --- | --- | --- | --- | --- |
| Temperature (TEM) | Meteorological factors | 1km | Aug 2022 | https://data.tpdc.ac.cn |
| Precipitation (PRE) |  | 1km | Aug 2022 |  |
| Relative humidity (RH) |  | 1km | Aug 2022 |  |
| Specific humidity (SH) |  | 1km | Aug 2022 |  |
| Potential evapotranspiration (PET) |  | 1km | Aug 2022 |  |
| Road networks density (Road) | Social factors | - | 2022 | https://www.openstreetmap.org |
| Land surface temperature (LST) |  | 1km | Aug 2022 | https://developers.google.cn/earth-engine/ |
| Population density (Pop) |  | 100m | 2020 | https://essd.copernicus.org/preprints/essd-2023-541/ |
| Regional gross domestic product (GDP) |  | 1km | 2022 | https://github.com/thestarlab/ChinaGDP |
| Intensity of night light (Night) |  | 500m | Aug 2022 | https://dataverse.harvard.edu |
| Distance to urban blue spaces (Distance) | Urban landscape | 30m | 2022 | https://zenodo.org/records/8176941 |
| Built areas density (Built) |  | 30m | 2022 | https://zenodo.org/records/8176941 |
| Urban green surface density (Green) |  | 30m | 2022 | https://zenodo.org/records/8176941 |
| Impervious surface area (GAIA) |  | 30m | 2022 | https://www.x-mol.com/groups/li_xuecao/dongtaizhitu |
| Normalized difference vegetation index (NDVI) |  | 250m | Aug 2022 | https://search.earthdata.nasa.gov/search |
| PM2.5 | Air pollution | 1km | Aug 2022 | https://data.tpdc.ac.cn |
| PM10 |  | 1km | Aug 2022 | https://data.tpdc.ac.cn |

**Note:** Meteorological factors include TEM, PRE, RH, SH, and PET. Temperature and humidity influence the growth [1–3], development, and oviposition cycle of *Aedes* mosquitoes, while changes in precipitation can affect the hatching process of mosquito eggs. Variations in potential evapotranspiration may be linked to changes in mosquito habitats and breeding environments [4], such as water-filled potholes and discarded containers in urban areas.

In terms of social factors are variables contain Road, LST, Pop, GDP, and Night. LST may directly influence the habitat preferences of *Aedes* mosquitoes [5]. The expansion of road networks, high population density, and frequent human activity provide more opportunities for blood-feeding and increase the availability of breeding sites for mosquito vectors [6,7]. Additionally, nighttime light exposurse can impact the core clock genes and microRNA regulatory mechanisms of *Aedes* mosquitoes, potentially enhancing their nocturnal blood-feeding behavior [8].

Urban landscape factors encompass Distance, Built, Green, GAIA, and NDVI. Changes in the quantity of urban blue and green spaces directly affect the number of *Aedes* mosquito breeding sites [9]. The distribution of urban buildings may influence mosquito flight paths [10], while the extent of impervious surfaces is associated with the availability of water-accumulating environments suitable for *Aedes* mosquito survival [11].

Air pollution factors include PM2.5 and PM10, Exposure to fine particulate matter can affect the blood-feeding rate and ovarian degeneration of *Aedes* mosquitoes [12].

**References:**

1. Mohammed A, Chadee DD. Effects of different temperature regimens on the development of *Aedes aegypti* (L.) (Diptera: Culicidae) mosquitoes. Acta Trop. 2011;119: 38–43. doi:10.1016/j.actatropica.2011.04.004

2. Sintorini MM. The correlation between temperature and humidity with the population density of Aedes aegypti as dengue fever’s vector. IOP Conf Ser Earth Environ Sci. 2018;106: 012033. doi:10.1088/1755-1315/106/1/012033

3. Valdez LD, Sibona GJ, Condat CA. Impact of rainfall on *Aedes aegypti* populations. Ecol Model. 2018;385: 96–105. doi:10.1016/j.ecolmodel.2018.07.003

4. Hayden MH, Uejio CK, Walker K, Ramberg F, Moreno R, Rosales C, et al. Microclimate and Human Factors in the Divergent Ecology of Aedes aegypti along the Arizona, U.S./Sonora, MX Border. EcoHealth. 2010;7: 64–77. doi:10.1007/s10393-010-0288-z

5. Moreno-Madriñán MJ, Crosson WL, Eisen L, Estes SM, Estes Jr. MG, Hayden M, et al. Correlating Remote Sensing Data with the Abundance of Pupae of the Dengue Virus Mosquito Vector, Aedes aegypti, in Central Mexico. ISPRS Int J Geo-Inf. 2014;3: 732–749. doi:10.3390/ijgi3020732

6. Bennett KL, Gómez Martínez C, Almanza A, Rovira JR, McMillan WO, Enriquez V, et al. High infestation of invasive Aedes mosquitoes in used tires along the local transport network of Panama. Parasit Vectors. 2019;12: 264. doi:10.1186/s13071-019-3522-8

7. Mahabir RS, Severson DW, Chadee DD. Impact of road networks on the distribution of dengue fever cases in Trinidad, West Indies. Acta Trop. 2012;123: 178–183. doi:10.1016/j.actatropica.2012.05.001

8. Rund SSC, Labb LF, Benefiel OM, Duffield GE. Artificial Light at Night Increases Aedes aegypti Mosquito Biting Behavior with Implications for Arboviral Disease Transmission. Am J Trop Med Hyg. 2020;103: 2450–2452. doi:10.4269/ajtmh.20-0885

9. Rhodes CG, Scavo NA, Finney M, Fimbres-Macias JP, Lively MT, Strauss BH, et al. Meta-Analysis of the Relative Abundance of Nuisance and Vector Mosquitoes in Urban and Blue-Green Spaces. Insects. 2022;13: 271. doi:10.3390/insects13030271

10. Liao J-R, Tu W-C, Chiu M-C, Kuo M-H, Cheng H-C, Chan C-C, et al. Joint influence of architectural and spatiotemporal factors on the presence of Aedes aegypti in urban environments. Pest Manag Sci. 2023;79: 4367–4375. doi:10.1002/ps.7634

11. Landau KI, van Leeuwen WJD. Fine scale spatial urban land cover factors associated with adult mosquito abundance and risk in Tucson, Arizona. J Vector Ecol. 2012;37: 407–418. doi:10.1111/j.1948-7134.2012.00245.x

12. Phanitchat T, Ampawong S, Yawootti A, Denpetkul T, Wadmanee N, Sompornrattanaphan M, et al. Dose-Dependent Blood-Feeding Activity and Ovarian Alterations to PM2.5 in Aedes aegypti. Insects. 2021;12: 948. doi:10.3390/insects12100948
