# Supplementary material for: Spatial pattern assessment of Aedes mosquito bite risk in a subtropical metropolitan area: A case study in Shenzhen
Source: PLoS Negl Trop Dis. 2025 Dec 23;19(12):e0013843. doi: 10.1371/journal.pntd.0013843 (PMC12725540; doi:10.1371/journal.pntd.0013843)
Supplement: S2 Table — (DOC) [file pntd.0013843.s006.doc]

**S2_Table. Specific spatial distribution of different population characteristics**

| Name | district | Lar_hou | Gender | Kid | Teenager | Elderly | One_hou | Low_edu | Flow | Small_hou | Low_inc | Agri_fish | High_den |
| --- | --- | --- | --- | --- | --- | --- | --- | --- | --- | --- | --- | --- | --- |
| Yanluo | Baoan | 1.81 | 151.85 | 3.31 | 5.83 | 1.31 | 50.17 | 7.64 | 74.60 | 46.16 | 8.59 | 0.12 | 5502.02 |
| Songgang | Baoan | 2.08 | 132.50 | 5.09 | 9.12 | 2.03 | 43.48 | 8.47 | 64.37 | 34.05 | 8.59 | 0.12 | 13130.61 |
| Xinqiao | Baoan | 2.08 | 132.68 | 5.20 | 8.54 | 1.70 | 42.48 | 7.86 | 61.44 | 34.53 | 8.59 | 0.12 | 13765.20 |
| Hangcheng | Baoan | 2.02 | 137.36 | 4.94 | 7.96 | 1.73 | 44.76 | 7.00 | 59.03 | 46.11 | 8.59 | 0.12 | 6774.10 |
| Fuyong | Baoan | 2.06 | 130.30 | 5.38 | 8.82 | 1.91 | 44.12 | 6.97 | 57.77 | 29.43 | 8.59 | 0.12 | 9262.89 |
| Shiyan | Baoan | 1.99 | 138.91 | 4.89 | 8.02 | 1.83 | 45.75 | 5.98 | 65.27 | 40.58 | 8.59 | 0.12 | 7834.30 |
| Xin’an | Baoan | 2.44 | 111.70 | 6.20 | 11.27 | 3.91 | 35.91 | 6.47 | 32.92 | 25.64 | 8.59 | 0.12 | 23895.36 |
| Xixiang | Baoan | 2.18 | 119.33 | 6.15 | 9.94 | 2.64 | 43.47 | 6.49 | 44.65 | 32.96 | 8.59 | 0.12 | 14398.53 |
| Shajin | Baoan | 2.01 | 133.90 | 4.70 | 7.86 | 1.78 | 43.65 | 6.77 | 66.23 | 37.71 | 8.59 | 0.12 | 15134.49 |
| Fuhai | Baoan | 1.92 | 139.16 | 4.08 | 6.98 | 1.32 | 47.00 | 5.24 | 65.32 | 40.51 | 8.59 | 0.12 | 13942.01 |
| Nanao | Dapeng | 2.70 | 134.32 | 6.65 | 11.53 | 6.71 | 30.24 | 20.30 | 28.33 | 13.93 | 21.83 | 0.94 | 145.51 |
| Dapeng | Dapeng | 2.32 | 146.04 | 5.82 | 9.60 | 3.91 | 39.97 | 11.27 | 47.38 | 23.32 | 21.83 | 0.94 | 743.21 |
| Kuiyong | Dapeng | 2.17 | 130.75 | 5.65 | 10.17 | 4.28 | 43.72 | 12.04 | 50.32 | 25.23 | 21.83 | 0.94 | 804.18 |
| Xiangmihu | Futian | 2.88 | 101.20 | 5.90 | 11.31 | 7.60 | 21.59 | 4.84 | 19.40 | 5.39 | 30.76 | 0.02 | 16680.59 |
| Futian | Futian | 2.44 | 107.86 | 5.93 | 9.88 | 3.85 | 37.78 | 7.23 | 31.94 | 24.61 | 30.76 | 0.02 | 34723.04 |
| Shatou | Futian | 2.17 | 109.68 | 4.69 | 7.93 | 3.36 | 42.59 | 5.77 | 40.83 | 35.16 | 30.76 | 0.02 | 21140.60 |
| Lianhua | Futian | 2.66 | 98.47 | 5.46 | 10.62 | 7.93 | 26.91 | 4.77 | 23.72 | 10.87 | 30.76 | 0.02 | 23487.83 |
| Meilin | Futian | 2.56 | 104.25 | 5.52 | 10.08 | 5.68 | 30.30 | 5.71 | 29.05 | 19.55 | 30.76 | 0.02 | 11635.48 |
| Huafu | Futian | 2.70 | 113.35 | 5.29 | 10.68 | 6.29 | 27.77 | 5.32 | 25.33 | 13.37 | 30.76 | 0.02 | 10622.89 |
| Nanyuan | Futian | 2.77 | 124.32 | 6.07 | 10.79 | 3.34 | 29.36 | 7.67 | 34.19 | 35.29 | 30.76 | 0.02 | 56844.43 |
| Huaqiangbei | Futian | 2.63 | 133.93 | 5.35 | 9.79 | 5.80 | 30.27 | 4.89 | 28.70 | 18.36 | 30.76 | 0.02 | 22653.60 |
| Yuanling | Futian | 2.69 | 106.90 | 5.30 | 11.60 | 7.82 | 26.53 | 4.93 | 26.80 | 11.02 | 30.76 | 0.02 | 33627.26 |
| Fubao | Futian | 2.62 | 99.02 | 5.75 | 11.03 | 6.68 | 27.68 | 4.62 | 25.66 | 11.54 | 30.76 | 0.02 | 25083.13 |
| Xinhu | Guangming | 1.96 | 160.15 | 4.27 | 7.39 | 1.82 | 46.56 | 11.05 | 66.79 | 35.00 | 10.29 | 0.30 | 2810.59 |
| Guangming | Guangming | 2.53 | 129.13 | 6.35 | 10.77 | 4.29 | 32.85 | 9.77 | 41.98 | 20.56 | 10.29 | 0.30 | 3692.51 |
| Matian | Guangming | 2.02 | 134.17 | 5.84 | 9.62 | 1.92 | 46.30 | 9.05 | 61.80 | 33.35 | 10.29 | 0.30 | 17163.49 |
| Gongming | Guangming | 2.02 | 132.39 | 5.25 | 9.00 | 1.84 | 46.21 | 9.60 | 65.58 | 33.22 | 10.29 | 0.30 | 8286.61 |
| Yutang | Guangming | 1.80 | 146.08 | 3.79 | 6.08 | 1.12 | 49.81 | 8.10 | 74.21 | 53.39 | 10.29 | 0.30 | 10472.82 |
| Fenghuang | Guangming | 1.93 | 156.69 | 4.73 | 7.33 | 1.56 | 47.67 | 10.06 | 68.46 | 37.89 | 10.29 | 0.30 | 7283.44 |
| Pingdi | Longgang | 1.94 | 138.35 | 4.76 | 8.94 | 2.25 | 51.23 | 11.82 | 61.73 | 36.79 | 11.99 | 0.11 | 4130.90 |
| Longgang | Longgang | 2.32 | 116.90 | 6.63 | 11.31 | 2.91 | 40.17 | 8.66 | 44.20 | 23.92 | 11.99 | 0.11 | 5074.42 |
| Baolong | Longgang | 2.24 | 133.63 | 5.61 | 9.57 | 2.26 | 40.66 | 9.35 | 56.03 | 27.63 | 11.99 | 0.11 | 9747.86 |
| Longcheng | Longgang | 2.61 | 108.93 | 7.36 | 12.69 | 4.29 | 31.91 | 7.29 | 32.58 | 17.58 | 11.99 | 0.11 | 13869.95 |
| Nanwan | Longgang | 2.36 | 115.18 | 6.82 | 10.71 | 3.45 | 38.72 | 8.17 | 38.41 | 21.66 | 11.99 | 0.11 | 15014.45 |
| Jihua | Longgang | 2.58 | 113.41 | 7.24 | 11.99 | 3.36 | 31.32 | 8.37 | 36.72 | 26.42 | 11.99 | 0.11 | 12341.46 |
| Bantian | Longgang | 2.08 | 120.29 | 6.48 | 9.65 | 2.47 | 47.68 | 5.45 | 46.30 | 31.99 | 11.99 | 0.11 | 20630.03 |
| Buji | Longgang | 2.66 | 104.14 | 7.27 | 12.37 | 4.48 | 29.93 | 7.44 | 29.43 | 20.58 | 11.99 | 0.11 | 34347.26 |
| Pinghu | Longgang | 2.21 | 129.51 | 5.12 | 8.89 | 2.13 | 39.74 | 9.82 | 54.91 | 38.02 | 11.99 | 0.11 | 10110.96 |
| Henggang | Longgang | 2.29 | 115.96 | 6.51 | 10.74 | 2.72 | 38.75 | 8.07 | 43.33 | 33.65 | 11.99 | 0.11 | 12147.36 |
| Yuanshan | Longgang | 2.11 | 134.06 | 4.78 | 9.08 | 1.97 | 43.18 | 9.13 | 57.16 | 36.91 | 11.99 | 0.11 | 5306.42 |
| Guanlan | Longhua | 1.97 | 142.85 | 4.20 | 7.51 | 1.96 | 46.56 | 8.23 | 64.26 | 38.90 | 9.85 | 0.08 | 6393.16 |
| Guanhu | Longhua | 2.21 | 134.22 | 5.88 | 9.79 | 2.35 | 41.57 | 7.15 | 54.77 | 31.27 | 9.85 | 0.08 | 9803.91 |
| Longhua | Longhua | 2.12 | 134.60 | 5.59 | 8.69 | 1.98 | 45.75 | 4.98 | 52.24 | 34.14 | 9.85 | 0.08 | 35881.95 |
| Fucheng | Longhua | 1.86 | 162.27 | 4.98 | 7.35 | 1.66 | 52.52 | 5.24 | 66.61 | 30.46 | 9.85 | 0.08 | 10996.88 |
| Dalang | Longhua | 2.08 | 131.93 | 5.47 | 8.98 | 1.79 | 44.68 | 6.04 | 58.65 | 39.52 | 9.85 | 0.08 | 12150.82 |
| Minzhi | Longhua | 2.34 | 108.56 | 7.43 | 11.01 | 3.30 | 38.96 | 5.25 | 39.46 | 22.03 | 9.85 | 0.08 | 21236.26 |
| Donghu | Luohu | 2.86 | 103.43 | 6.48 | 12.94 | 5.97 | 23.04 | 7.66 | 26.46 | 12.65 | 20.64 | 0.08 | 3826.86 |
| Nanhu | Luohu | 2.05 | 94.12 | 4.43 | 7.88 | 3.97 | 48.92 | 5.96 | 42.13 | 22.02 | 20.64 | 0.08 | 34923.86 |
| Huangbei | Luohu | 2.73 | 103.55 | 6.01 | 11.26 | 5.68 | 27.63 | 7.55 | 26.63 | 19.62 | 20.64 | 0.08 | 25621.36 |
| Dongxiao | Luohu | 3.09 | 111.84 | 5.95 | 12.59 | 4.61 | 19.20 | 8.11 | 24.00 | 22.93 | 20.64 | 0.08 | 33018.12 |
| Liantang | Luohu | 2.55 | 100.60 | 5.76 | 11.09 | 5.96 | 29.18 | 5.53 | 31.06 | 20.47 | 20.64 | 0.08 | 10638.93 |
| Guiyuan | Luohu | 2.43 | 98.55 | 4.52 | 8.59 | 7.01 | 36.52 | 4.58 | 31.80 | 11.94 | 20.64 | 0.08 | 40823.46 |
| Qingshuihe | Luohu | 2.61 | 118.07 | 5.42 | 10.15 | 4.54 | 28.07 | 8.68 | 38.92 | 27.96 | 20.64 | 0.08 | 8851.26 |
| Cuihu | Luohu | 2.65 | 99.24 | 5.78 | 11.08 | 6.53 | 28.50 | 6.15 | 25.77 | 14.51 | 20.64 | 0.08 | 39033.79 |
| Dongmen | Luohu | 2.71 | 102.76 | 5.36 | 10.85 | 5.01 | 30.32 | 9.35 | 28.34 | 32.98 | 20.64 | 0.08 | 43291.07 |
| Sungang | Luohu | 2.45 | 108.65 | 6.01 | 10.48 | 4.76 | 33.44 | 7.21 | 30.28 | 27.59 | 20.64 | 0.08 | 26427.64 |
| Xili | Nanshan | 2.18 | 127.14 | 4.75 | 7.88 | 2.60 | 42.43 | 5.38 | 45.57 | 28.69 | 36.35 | 0.12 | 6715.83 |
| Yuehai | Nanshan | 2.63 | 111.29 | 5.04 | 9.73 | 5.46 | 27.94 | 3.79 | 26.00 | 6.83 | 36.35 | 0.12 | 18442.76 |
| Nantou | Nanshan | 2.58 | 111.21 | 5.17 | 9.78 | 5.11 | 30.05 | 5.16 | 34.45 | 21.00 | 36.35 | 0.12 | 17785.34 |
| Zhaoshang | Nanshan | 2.71 | 111.30 | 5.29 | 10.13 | 6.46 | 23.97 | 4.26 | 26.46 | 6.60 | 36.35 | 0.12 | 8375.41 |
| Shekou | Nanshan | 2.60 | 106.61 | 5.83 | 10.60 | 5.04 | 28.14 | 6.86 | 32.66 | 23.41 | 36.35 | 0.12 | 12432.20 |
| Nanshan | Nanshan | 2.52 | 111.23 | 5.57 | 9.61 | 4.23 | 31.19 | 5.75 | 39.29 | 21.79 | 36.35 | 0.12 | 13076.71 |
| Shahe | Nanshan | 2.68 | 106.61 | 4.76 | 8.87 | 6.41 | 25.83 | 5.68 | 32.15 | 14.93 | 36.35 | 0.12 | 10127.90 |
| Taoyuan | Nanshan | 2.41 | 118.77 | 5.80 | 9.42 | 3.99 | 36.02 | 4.94 | 35.75 | 18.71 | 36.35 | 0.12 | 6255.33 |
| Shijin | Pingshan | 2.00 | 175.28 | 3.71 | 6.57 | 2.25 | 48.02 | 13.70 | 53.75 | 37.61 | 14.61 | 0.38 | 1255.98 |
| Longtian | Pingshan | 1.94 | 138.43 | 5.26 | 8.67 | 1.93 | 49.15 | 9.76 | 62.59 | 34.51 | 14.61 | 0.38 | 3737.61 |
| Kengzi | Pingshan | 2.15 | 134.77 | 5.80 | 10.24 | 2.33 | 44.36 | 11.19 | 57.85 | 36.53 | 14.61 | 0.38 | 5430.87 |
| Pingshan | Pingshan | 2.49 | 121.87 | 7.15 | 12.78 | 3.15 | 35.08 | 10.41 | 44.37 | 29.42 | 14.61 | 0.38 | 11256.30 |
| Maluan | Pingshan | 2.05 | 147.11 | 6.30 | 10.28 | 2.53 | 50.07 | 8.47 | 55.05 | 29.80 | 14.61 | 0.38 | 2153.25 |
| Biling | Pingshan | 2.14 | 137.31 | 5.55 | 10.05 | 2.64 | 44.66 | 13.28 | 56.98 | 35.35 | 14.61 | 0.38 | 2440.76 |
| Shatoujiao | Yantian | 2.84 | 106.95 | 5.71 | 10.82 | 5.89 | 25.03 | 7.79 | 24.19 | 18.00 | 30.37 | 0.03 | 6561.64 |
| Haishan | Yantian | 2.88 | 115.09 | 5.72 | 10.26 | 5.83 | 23.34 | 5.45 | 28.30 | 15.94 | 30.37 | 0.03 | 8638.32 |
| Meisha | Yantian | 2.39 | 132.48 | 4.54 | 8.68 | 4.84 | 34.70 | 8.43 | 40.37 | 18.14 | 30.37 | 0.03 | 1053.62 |
| Yantian | Yantian | 2.79 | 125.25 | 5.63 | 10.79 | 3.27 | 24.95 | 6.96 | 39.95 | 30.38 | 30.37 | 0.03 | 2033.67 |
