# Supplementary material for: Spatial pattern assessment of Aedes mosquito bite risk in a subtropical metropolitan area: A case study in Shenzhen
Source: PLoS Negl Trop Dis. 2025 Dec 23;19(12):e0013843. doi: 10.1371/journal.pntd.0013843 (PMC12725540; doi:10.1371/journal.pntd.0013843)
Supplement: S3 Table — (DOC) [file pntd.0013843.s007.doc]

**S3_Table.** Pearson correlation coefficients between the number of dengue fever cases and risk assessment components

| Time period | Factors | r | *p*-value |
| --- | --- | --- | --- |
| August 2022 | IMD score | 0.107 | 0.36 |
|  | MOI | 0.242 | <0.05 |
|  | HH hazard-exposure score | 0.412 | <0.05 |
|  | Composite risk score | **0.446** | <0.05 |
| September 2022 | IMD score | 0.162 | 0.17 |
|  | MOI | 0.120 | 0.30 |
|  | HH hazard-exposure score | 0.388 | <0.05 |
|  | Composite risk score | **0.397** | <0.05 |
| October 2022 | IMD score | 0.125 | 0.28 |
|  | MOI | -0.115 | 0.32 |
|  | HH hazard-exposure score | 0.402 | <0.05 |
|  | Composite risk score | **0.406** | <0.05 |

To validate the epidemiological relevance of our risk assessment framework, we examined the correlation between framework-derived risk components and the number of dengue fever cases reported across the 74 subdistricts during August-October 2022 (S3_Table). The analysis revealed that the composite risk score demonstrated the strongest and most consistent correlation with dengue cases across all three months (r = 0.446, *p* < 0.05 in August; r = 0.397, *p* < 0.05 in September; r = 0.406, *p* < 0.05 in October), confirming that the integrated framework successfully captures disease transmission patterns.

Among individual components, the HH hazard-exposure score showed consistently significant positive correlations with dengue cases (r = 0.388-0.412, all p < 0.05), indicating that areas where high mosquito density and high human population spatially converge are indeed at elevated risk for disease transmission. In contrast, MOI alone showed weak or non-significant correlations in September and October (r = 0.120 and -0.115, both p > 0.05), with a counterintuitive negative correlation emerging in October. This suggests that *Aedes* mosquito presence without considering human exposure provides limited predictive value for disease occurrence. The IMD (vulnerability) component showed no significant correlation with dengue cases in any month, indicating that while vulnerability influences population susceptibility, it does not independently predict disease occurrence without corresponding hazard and exposure.

These findings validate three key aspects of our framework. Firstly, the superiority of integrated risk assessment over single-component approaches. Secondly, the critical importance of spatial co-occurrence between mosquito hazard and human exposure for disease transmission. Additionally, the framework showed stable predictive performance across different months of the transmission season. The consistent positive correlations suggest that our risk maps can effectively guide targeted surveillance and intervention efforts.
