# Supplementary figures and images for: Spatial pattern assessment of Aedes mosquito bite risk in a subtropical metropolitan area: A case study in Shenzhen
Source: PLoS Negl Trop Dis. 2025 Dec 23;19(12):e0013843. doi: 10.1371/journal.pntd.0013843 (PMC12725540; doi:10.1371/journal.pntd.0013843)

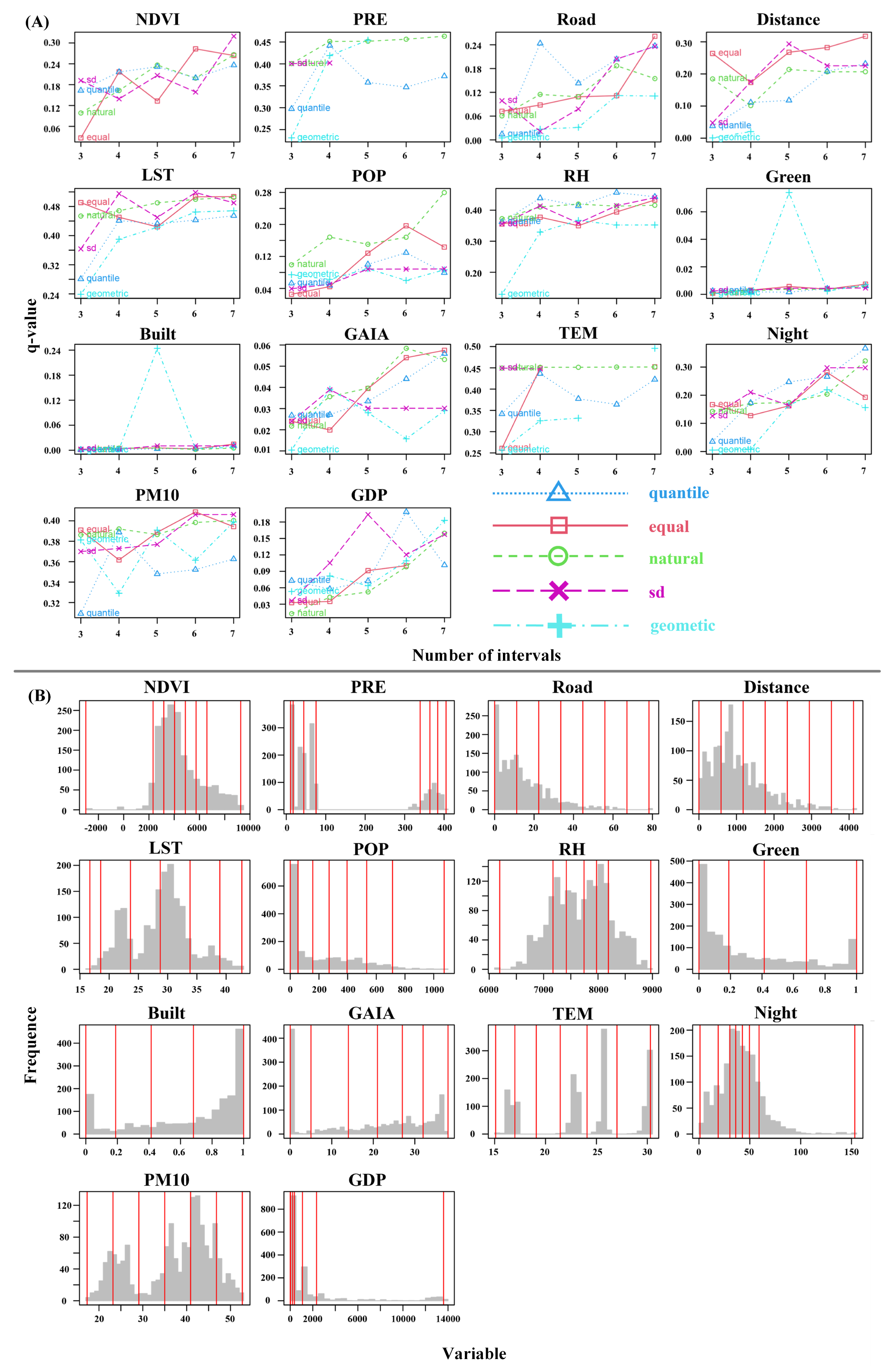

Supplement: S1 Fig — (TIF) [file pntd.0013843.s008.tif]

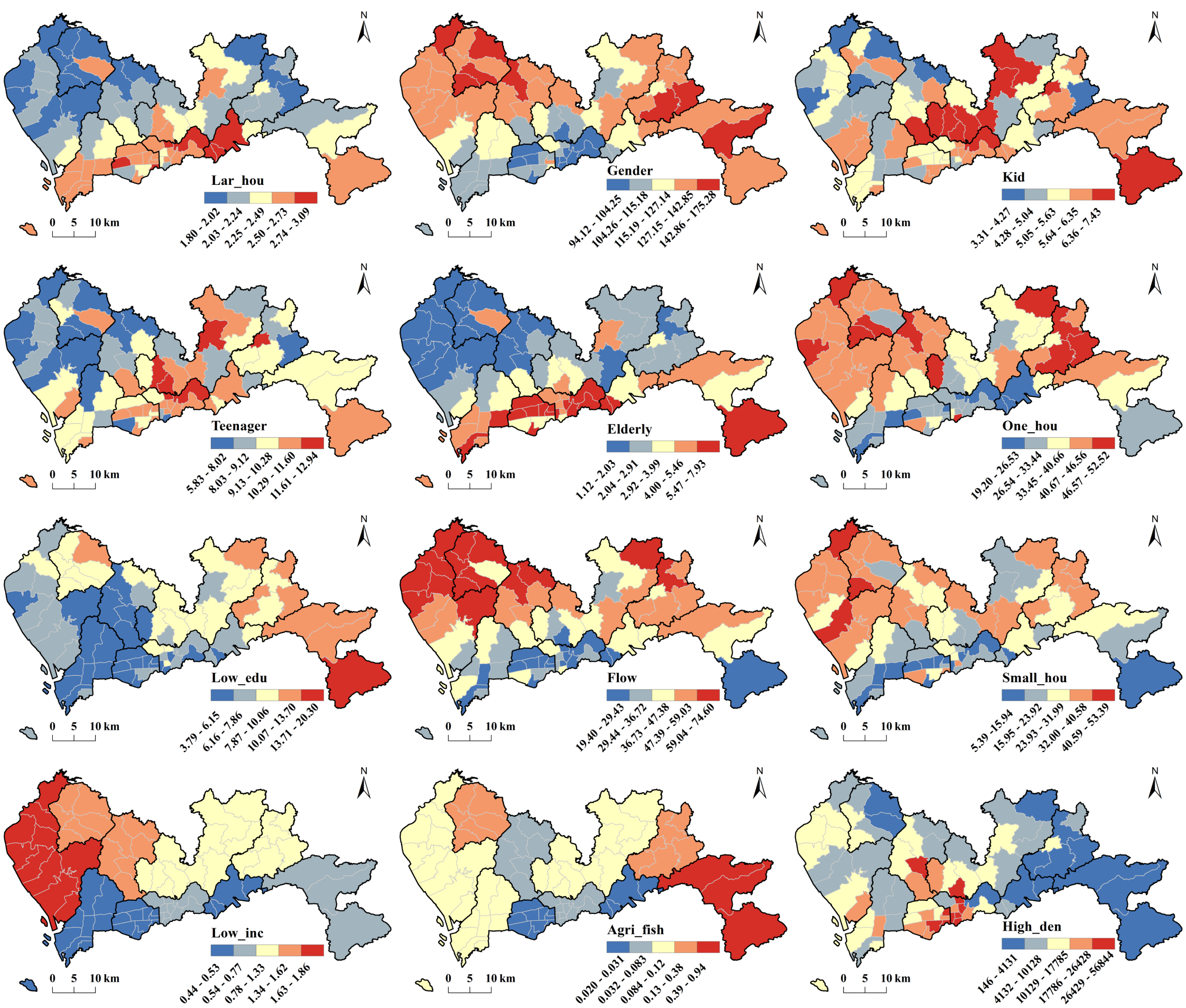

Supplement: S2 Fig — Base map credit: This figure uses the standard map (Approval Number: GS(2023)2767) supervised by the Ministry of Natural Resources of the People’s Republic of China (http://bzdt.ch.mnr.gov.cn/). The boundary of the base map has not been modified. (TIF) [file pntd.0013843.s009.tif]

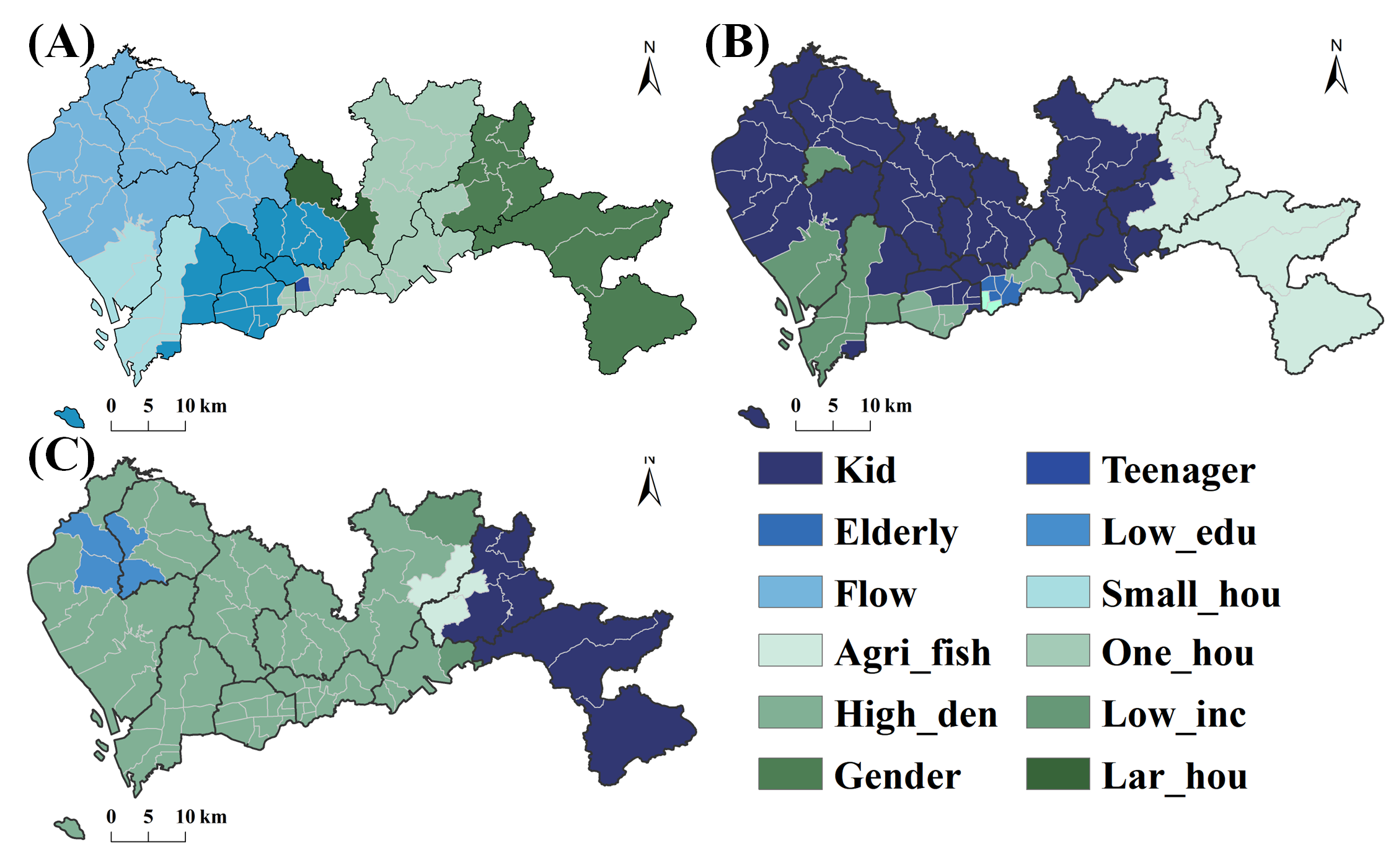

Supplement: S3 Fig — (A) GWPC1; (B) GWPC2; (C) GWPC3. Base map credit: This figure uses the standard map (Approval Number: GS(2023)2767) supervised by the Ministry of Natural Resources of the People’s Republic of China (http://bzdt.ch.mnr.gov.cn/). The boundary of the base map has not been modified. (TIF) [file pntd.0013843.s010.tif]

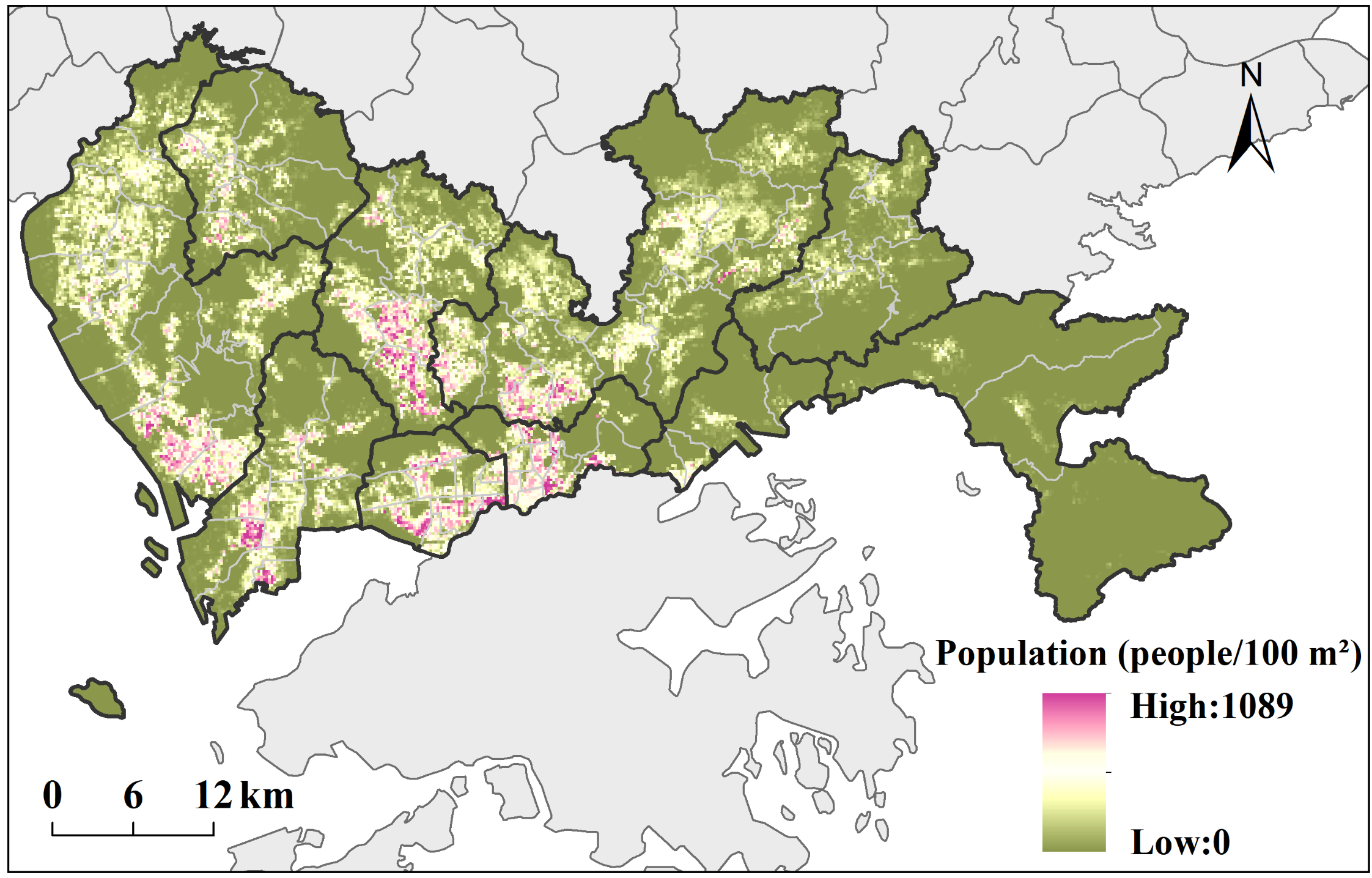

Supplement: S4 Fig — Base map credit: This figure uses the standard map (Approval Number: GS(2023)2767) supervised by the Ministry of Natural Resources of the People’s Republic of China (http://bzdt.ch.mnr.gov.cn/). The boundary of the base map has not been modified. (TIF) [file pntd.0013843.s011.tif]

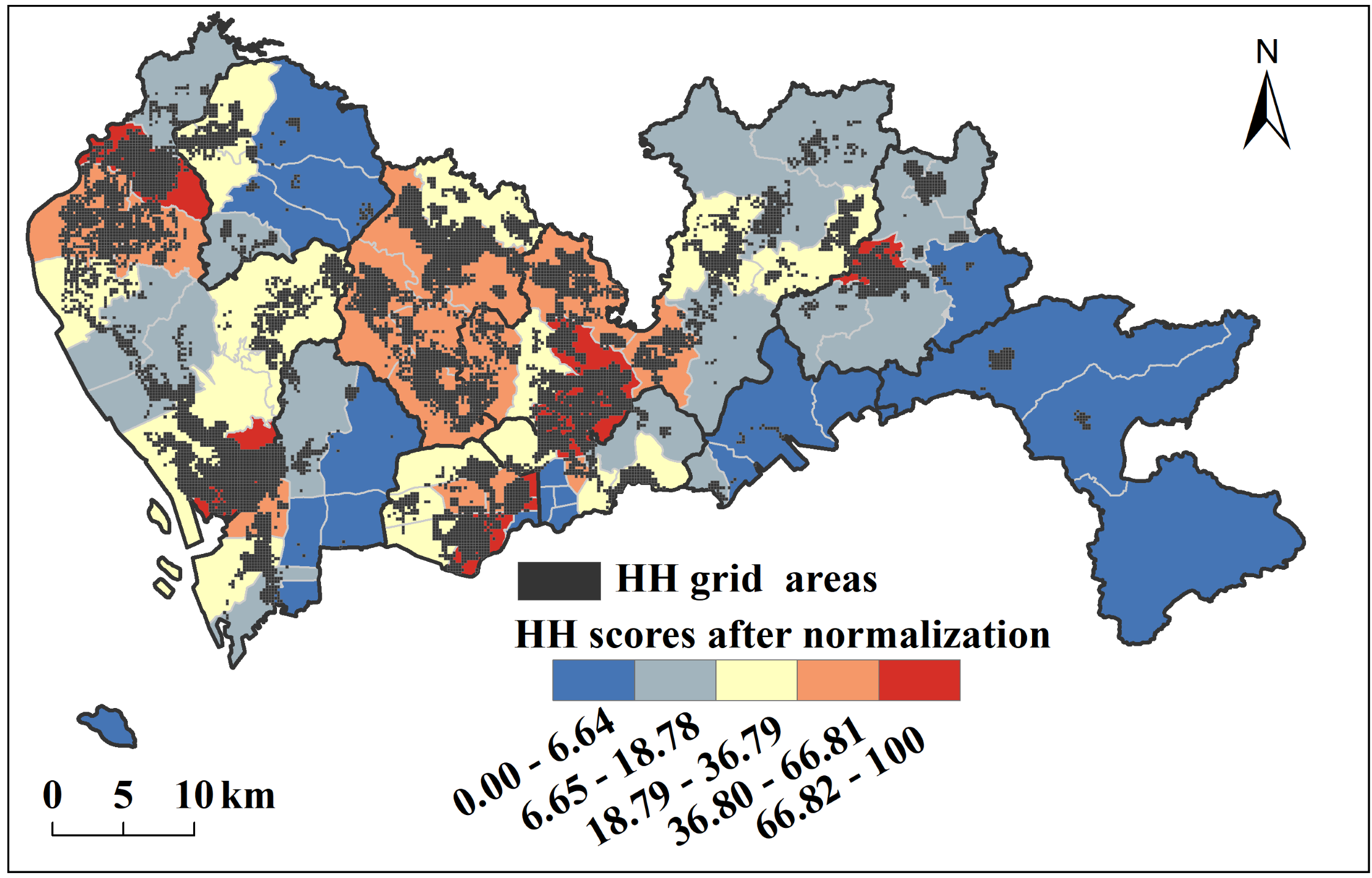

Supplement: S5 Fig — Base map credit: This figure uses the standard map (Approval Number: GS(2023)2767) supervised by the Ministry of Natural Resources of the People’s Republic of China (http://bzdt.ch.mnr.gov.cn/). The boundary of the base map has not been modified. (TIF) [file pntd.0013843.s012.tif]

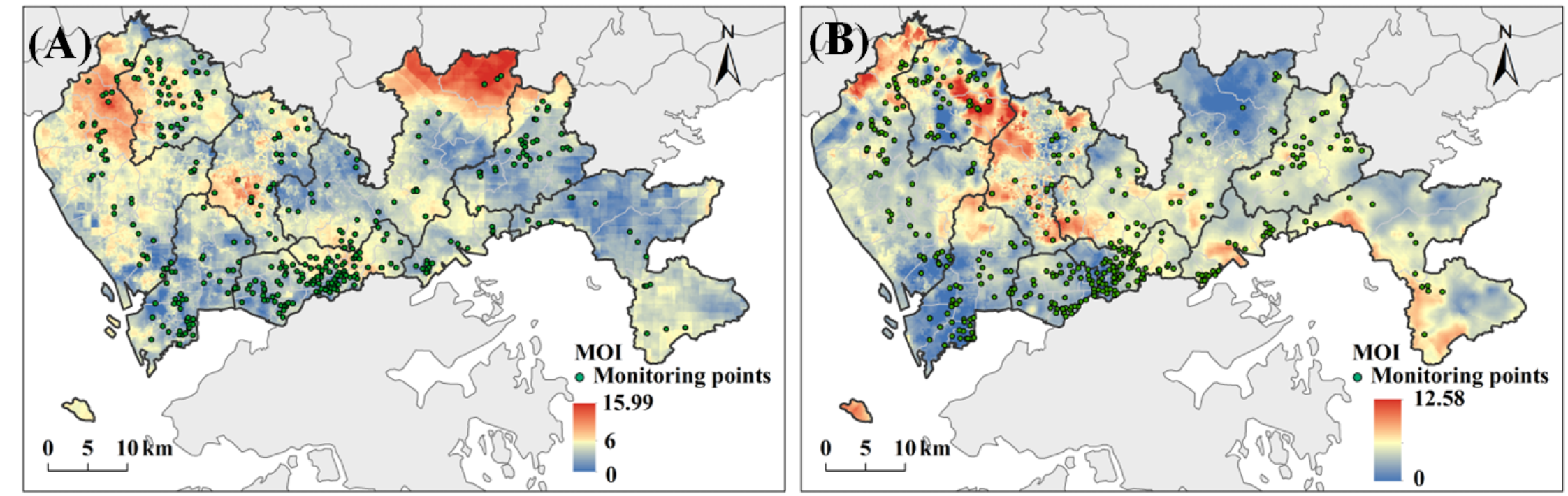

Supplement: S6 Fig — (A) The MOI map in September, 2022; (B) The MOI map in October, 2022 (200m spatial resolution). Base map credit: This figure uses the standard map (Approval Number: GS(2023)2767) supervised by the Ministry of Natural Resources of the People’s Republic of China (http://bzdt.ch.mnr.gov.cn/). The boundary of the base map has not been modified. (TIF) [file pntd.0013843.s013.tif]

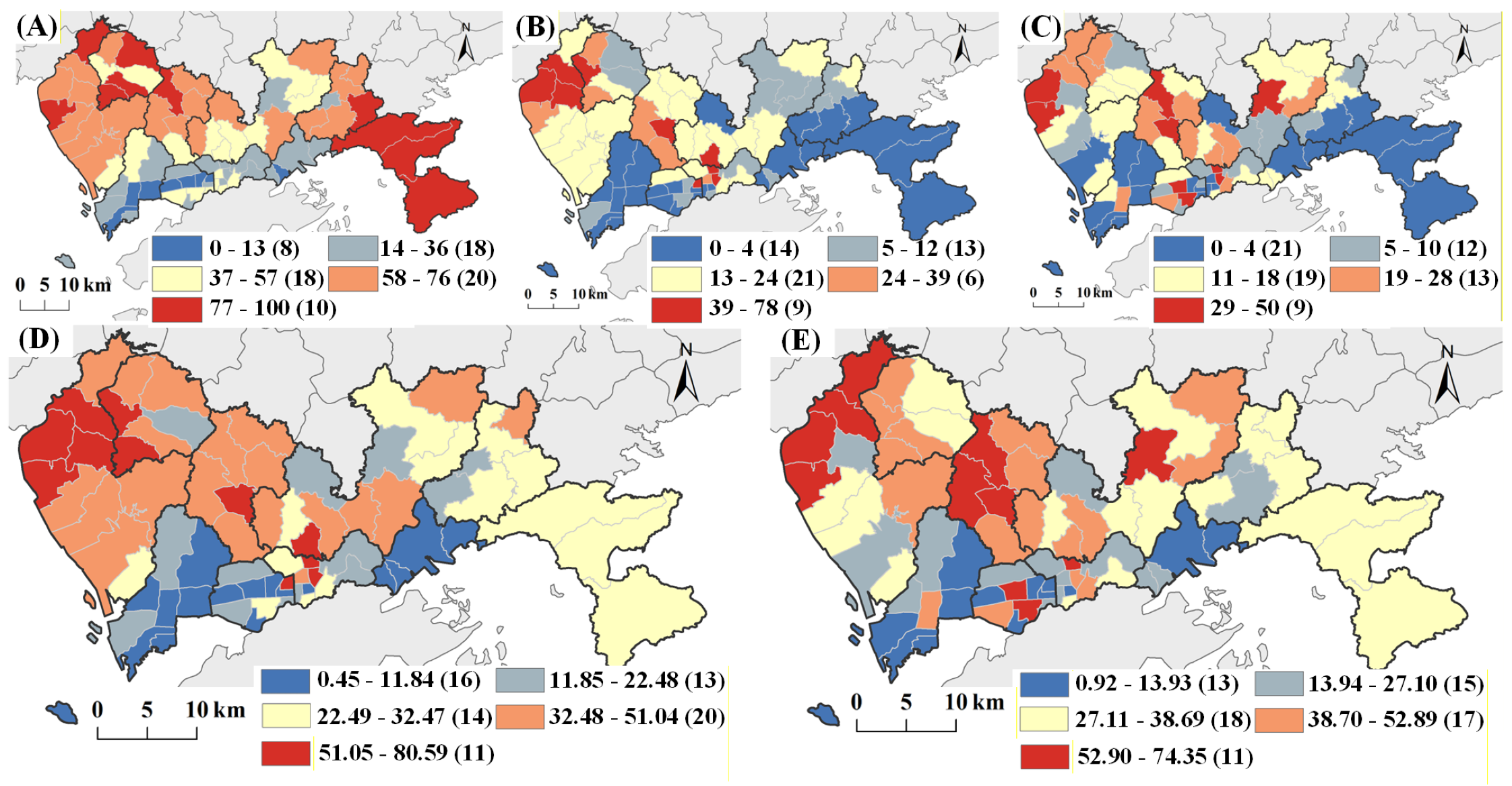

Supplement: S7 Fig — (A) Spatial pattern of vulnerability; (B) Local spatial association of HH hazard-exposure in September; (C) Local spatial association of HH hazard-exposure in October; (D) Aedes mosquito bite risk assessment map in September; (E) Aedes mosquito bite risk assessment map in October. Base map credit: This figure uses the standard map (Approval Number: GS(2023)2767) supervised by the Ministry of Natural Resources of the People’s Republic of China (http://bzdt.ch.mnr.gov.cn/). The boundary of the base map has not been modified. (TIF) [file pntd.0013843.s014.tif]
