# Supplementary material for: Spatial pattern assessment of Aedes mosquito bite risk in a subtropical metropolitan area: A case study in Shenzhen
Source: PLoS Negl Trop Dis. 2025 Dec 23;19(12):e0013843. doi: 10.1371/journal.pntd.0013843 (PMC12725540; doi:10.1371/journal.pntd.0013843)
Supplement: S1 Data — (DOCX) [file pntd.0013843.s015.docx]

**Table 1. Original data of MOI**

| **MOI** | **X** | **Y** | **MOI** | **X** | **Y** |
| --- | --- | --- | --- | --- | --- |
| 18 | 114.0692 | 22.6208 | 18.18 | 114.0219 | 22.6349 |
| 7.55 | 114.0636 | 22.6388 | 7.14 | 114.1131 | 22.5377 |
| 19.23 | 114.0574 | 22.6230 | 5.36 | 114.1132 | 22.5391 |
| 2.94 | 114.0687 | 22.6424 | 21.26 | 113.9107 | 22.5184 |
| 13.64 | 114.0835 | 22.6517 | 22.64 | 113.9105 | 22.5185 |
| 9.26 | 114.0753 | 22.6454 | 18.33 | 113.9103 | 22.5077 |
| 7.14 | 114.3147 | 22.6946 | 13.77 | 113.9127 | 22.5236 |
| 1.55 | 114.2742 | 22.6814 | 16.9 | 113.9132 | 22.5232 |
| 10.91 | 114.3161 | 22.6976 | 7.02 | 113.8993 | 22.5003 |
| 3.51 | 114.3098 | 22.6963 | 15.17 | 113.9145 | 22.5312 |
| 7.27 | 114.3132 | 22.7142 | 16.27 | 113.9129 | 22.5297 |
| 1.72 | 114.3091 | 22.7170 | 15.45 | 113.9229 | 22.5306 |
| 12.57 | 114.2871 | 22.6622 | 11.9 | 113.9119 | 22.5448 |
| 11.75 | 114.2883 | 22.6611 | 12.77 | 113.9190 | 22.5478 |
| 3.51 | 114.3121 | 22.6705 | 12.65 | 113.9241 | 22.5455 |
| 15.79 | 114.2844 | 22.6599 | 6 | 114.1572 | 22.6275 |
| 3.64 | 114.3192 | 22.6731 | 8.51 | 114.1388 | 22.6088 |
| 9.8 | 114.1078 | 22.5925 | 3.92 | 114.1454 | 22.6099 |
| 9.09 | 114.1224 | 22.6062 | 8.16 | 114.1451 | 22.5976 |
| 7.14 | 114.1362 | 22.6000 | 5.88 | 114.1643 | 22.6081 |
| 9.26 | 114.1303 | 22.6070 | 7.27 | 114.1053 | 22.5373 |
| 9.26 | 114.1192 | 22.5933 | 5.88 | 114.1048 | 22.5377 |
| 9.26 | 114.1309 | 22.6183 | 7.14 | 114.0935 | 22.5394 |
| 5.26 | 114.1322 | 22.5731 | 8.55 | 114.1035 | 22.5398 |
| 5.17 | 114.1343 | 22.5687 | 3.57 | 114.1552 | 22.6502 |
| 8.93 | 114.1303 | 22.5632 | 2.27 | 114.1548 | 22.6514 |
| 12.92 | 114.1254 | 22.5610 | 3.7 | 114.1545 | 22.6516 |
| 3.64 | 114.1280 | 22.5575 | 5.66 | 114.1511 | 22.6632 |
| 7.02 | 114.1361 | 22.5739 | 7.27 | 114.1517 | 22.6621 |
| 15.25 | 114.0102 | 22.6448 | 3.45 | 114.1424 | 22.6673 |
| 7.02 | 114.0091 | 22.6405 | 8.77 | 114.3193 | 22.7774 |
| 13.56 | 114.0097 | 22.6392 | 8.77 | 114.2842 | 22.7586 |
| 15.52 | 113.9651 | 22.6515 | 8.93 | 114.3070 | 22.7803 |
| 12.5 | 113.9361 | 22.6615 | 10.91 | 114.2890 | 22.7538 |
| 9.09 | 113.9943 | 22.6649 | 10.91 | 114.3163 | 22.7805 |
| 3.64 | 114.1443 | 22.5787 | 9.26 | 114.2944 | 22.7504 |
| 6.32 | 114.1430 | 22.5785 | 12.5 | 114.1073 | 22.5730 |
| 7.41 | 114.1719 | 22.6031 | 8.93 | 114.0892 | 22.5722 |
| 3.45 | 114.1750 | 22.6001 | 7.41 | 114.0990 | 22.5689 |
| 2.35 | 114.1403 | 22.5775 | 5.45 | 114.1028 | 22.5733 |
| 9.26 | 114.1914 | 22.5906 | 7.02 | 114.0871 | 22.5755 |
| 1.72 | 114.1214 | 22.5476 | 3.57 | 114.0998 | 22.5663 |
| 9.26 | 114.1243 | 22.5540 | 9.68 | 113.9908 | 22.5373 |
| 3.64 | 114.1185 | 22.5508 | 11.71 | 113.9935 | 22.5373 |
| 5.26 | 114.1172 | 22.5532 | 9.34 | 113.9904 | 22.5420 |
| 4.62 | 114.1204 | 22.5536 | 7.2 | 113.9877 | 22.5422 |
| 6.9 | 114.1222 | 22.5551 | 12.79 | 113.9868 | 22.5408 |
| 3.51 | 114.1199 | 22.5835 | 18.97 | 113.8084 | 22.7137 |
| 5.56 | 114.1325 | 22.5850 | 5.36 | 113.8059 | 22.7144 |
| 1.58 | 114.1354 | 22.5814 | 9.09 | 113.7707 | 22.7270 |
| 8.77 | 114.1231 | 22.5855 | 12.28 | 113.7782 | 22.7357 |
| 8.77 | 114.1372 | 22.5832 | 5.08 | 113.8053 | 22.7322 |
| 8.47 | 114.1276 | 22.5825 | 5.36 | 113.8064 | 22.7326 |
| 5.17 | 113.9213 | 22.7316 | 9.54 | 114.2208 | 22.5568 |
| 7.02 | 113.9212 | 22.7315 | 9.43 | 114.2206 | 22.5567 |
| 3.33 | 113.9135 | 22.7270 | 7.84 | 114.0330 | 22.5184 |
| 5.56 | 113.9134 | 22.7270 | 10.27 | 114.0360 | 22.5260 |
| 5.36 | 113.9050 | 22.7504 | 5.17 | 114.0230 | 22.5330 |
| 9.43 | 113.9050 | 22.7504 | 9.05 | 114.0347 | 22.5206 |
| 12.54 | 114.0551 | 22.5215 | 8.76 | 114.0420 | 22.5188 |
| 6.9 | 114.0559 | 22.5147 | 7.41 | 113.8915 | 22.4973 |
| 12.65 | 114.0519 | 22.5228 | 5.77 | 113.9258 | 22.4918 |
| 8.75 | 114.0487 | 22.5185 | 7.14 | 113.9055 | 22.4941 |
| 14.55 | 114.0776 | 22.5503 | 5.36 | 113.9304 | 22.4874 |
| 9.43 | 114.0503 | 22.5143 | 7.27 | 113.9311 | 22.4849 |
| 19.26 | 114.0400 | 22.7112 | 12.5 | 113.9433 | 22.6857 |
| 20.69 | 114.0447 | 22.7164 | 9.26 | 113.9477 | 22.6800 |
| 25 | 114.0461 | 22.7154 | 10.34 | 113.9366 | 22.6759 |
| 8.62 | 114.0223 | 22.6558 | 5.26 | 113.9379 | 22.6747 |
| 8.77 | 114.0223 | 22.6559 | 7.27 | 113.9372 | 22.6781 |
| 18.97 | 114.0421 | 22.6945 | 7.84 | 113.9407 | 22.6782 |
| 8.93 | 113.8314 | 22.6920 | 9.09 | 113.8522 | 22.7632 |
| 8.93 | 113.8126 | 22.7043 | 8.47 | 113.8304 | 22.7644 |
| 3.57 | 113.8280 | 22.6975 | 5.26 | 113.8160 | 22.7753 |
| 5.26 | 113.8303 | 22.7024 | 7.14 | 113.8132 | 22.7575 |
| 7.02 | 113.8125 | 22.7026 | 3.64 | 113.8404 | 22.7666 |
| 16.46 | 114.0512 | 22.5316 | 7.41 | 113.8409 | 22.6301 |
| 9.26 | 114.0766 | 22.5319 | 3.69 | 114.1068 | 22.5618 |
| 8.93 | 114.0580 | 22.5176 | 5.45 | 114.1238 | 22.5577 |
| 9.26 | 114.0751 | 22.5258 | 8.93 | 114.1225 | 22.5691 |
| 5.27 | 114.0678 | 22.5210 | 8.62 | 114.1182 | 22.5641 |
| 7.69 | 114.0722 | 22.5377 | 7.02 | 114.1202 | 22.5588 |
| 5.17 | 113.8387 | 22.6869 | 2.97 | 114.1120 | 22.5641 |
| 5.08 | 113.8339 | 22.6915 | 5.77 | 113.9915 | 22.5904 |
| 8.77 | 113.8206 | 22.6780 | 5.56 | 113.9863 | 22.5624 |
| 6.78 | 113.8163 | 22.6736 | 5.45 | 114.0048 | 22.5942 |
| 5.17 | 113.8278 | 22.6749 | 5.77 | 113.9875 | 22.5944 |
| 8.51 | 113.8893 | 22.7846 | 8.62 | 113.9803 | 22.5625 |
| 9.62 | 113.8869 | 22.8047 | 5.17 | 113.9938 | 22.5712 |
| 9.62 | 113.8869 | 22.8047 | 5.66 | 113.9517 | 22.5673 |
| 8 | 113.9053 | 22.7844 | 5.66 | 113.9497 | 22.5830 |
| 9.8 | 113.9053 | 22.7845 | 7.27 | 113.9494 | 22.5797 |
| 15.09 | 114.0699 | 22.7164 | 9.09 | 113.9505 | 22.5783 |
| 15.26 | 114.0699 | 22.7164 | 8.93 | 113.9473 | 22.5586 |
| 10.14 | 114.0639 | 22.6931 | 16.64 | 113.9326 | 22.5737 |
| 10.54 | 114.0475 | 22.6849 | 3.45 | 113.8710 | 22.6061 |
| 12.41 | 114.0475 | 22.6849 | 16.36 | 113.8530 | 22.5840 |
| 20.69 | 114.0475 | 22.6929 | 15.79 | 113.8600 | 22.5768 |
| 12.09 | 114.0377 | 22.7417 | 8.62 | 113.8518 | 22.5764 |
| 11.77 | 114.0377 | 22.7419 | 5.45 | 113.8765 | 22.6083 |
| 11.62 | 114.0522 | 22.7463 | 8.93 | 113.8642 | 22.6159 |
| 9.9 | 114.0534 | 22.7461 | 3.39 | 114.0263 | 22.5388 |
| 12.09 | 114.0938 | 22.7315 | 6.9 | 114.0181 | 22.5441 |
| 11.47 | 114.0958 | 22.7329 | 6.78 | 114.0301 | 22.5549 |
| 2.44 | 113.9544 | 22.7504 | 5.17 | 114.0216 | 22.5554 |
| 2.38 | 113.9547 | 22.7499 | 6.78 | 114.0192 | 22.5512 |
| 11.63 | 113.9238 | 22.7626 | 19.09 | 113.9149 | 22.5622 |
| 15.91 | 113.9236 | 22.7627 | 19.25 | 113.9050 | 22.5581 |
| 2.5 | 113.9431 | 22.7603 | 36.43 | 113.9117 | 22.5651 |
| 2.33 | 113.9431 | 22.7603 | 25.93 | 113.9164 | 22.5701 |
| 3.7 | 114.1137 | 22.5556 | 19.65 | 113.9135 | 22.5576 |
| 9.26 | 114.1142 | 22.5556 | 12.93 | 113.8961 | 22.5443 |
| 3.45 | 114.1099 | 22.5496 | 2.5 | 113.9184 | 22.7780 |
| 3.7 | 114.1138 | 22.5518 | 9.8 | 113.9193 | 22.7778 |
| 7.27 | 114.1117 | 22.5474 | 8.7 | 113.9564 | 22.7415 |
| 1.79 | 113.9086 | 22.7691 | 13.04 | 113.9560 | 22.7416 |
| 9.28 | 114.2393 | 22.5607 | 3.64 | 113.9613 | 22.7900 |
| 11.19 | 114.2387 | 22.5601 | 2.04 | 113.9602 | 22.7895 |
| 8.77 | 114.1277 | 22.5601 | 8.77 | 113.8361 | 22.7165 |
| 6.9 | 113.8376 | 22.6335 | 7.02 | 113.8359 | 22.7212 |
| 14.04 | 113.8417 | 22.6297 | 3.57 | 113.8283 | 22.7229 |
| 14.04 | 113.8635 | 22.6410 | 15.52 | 113.8310 | 22.7237 |
| 8.93 | 113.8639 | 22.6484 | 10.34 | 113.8399 | 22.7346 |
| 8.77 | 114.2020 | 22.6407 | 7.02 | 114.2605 | 22.5874 |
| 8.93 | 114.2093 | 22.6466 | 3.57 | 114.2532 | 22.5898 |
| 7.27 | 114.2088 | 22.6463 | 7.55 | 114.2414 | 22.5853 |
| 7.14 | 114.1735 | 22.6435 | 7.02 | 113.8425 | 22.7838 |
| 3.45 | 114.1287 | 22.6354 | 13.79 | 113.8910 | 22.8330 |
| 7.41 | 114.1861 | 22.6475 | 7.14 | 113.8521 | 22.7730 |
| 8.47 | 114.0809 | 22.5629 | 6.9 | 113.8486 | 22.7840 |
| 10.9 | 114.0817 | 22.5514 | 5.26 | 113.8500 | 22.7974 |
| 10.71 | 114.0670 | 22.5377 | 9.26 | 113.8583 | 22.7730 |
| 15.25 | 114.0763 | 22.5457 | 3.39 | 113.9065 | 22.7075 |
| 9.87 | 114.0807 | 22.5513 | 6.78 | 113.9066 | 22.7074 |
| 6.67 | 113.9005 | 22.4993 | 7.27 | 113.9132 | 22.7266 |
| 7.06 | 114.0940 | 22.5473 | 10.71 | 113.9130 | 22.7265 |
| 8.87 | 114.0902 | 22.5482 | 5.45 | 113.9002 | 22.7199 |
| 15.52 | 114.0845 | 22.5502 | 8.62 | 113.9002 | 22.7198 |
| 8.47 | 114.1023 | 22.5455 | 6.78 | 114.0966 | 22.5497 |
| 6.87 | 114.0972 | 22.5477 | 8.33 | 114.0926 | 22.5500 |
| 5.08 | 114.0946 | 22.5422 | 5 | 114.0984 | 22.5527 |
| 5.36 | 114.1417 | 22.5639 | 6.9 | 114.1005 | 22.5542 |
| 7.02 | 114.1441 | 22.5588 | 8.65 | 114.0976 | 22.5504 |
| 13.89 | 114.1523 | 22.5624 | 9.08 | 114.0884 | 22.5508 |
| 1.75 | 114.1352 | 22.5519 | 3.57 | 114.2194 | 22.6308 |
| 3.45 | 114.1420 | 22.5526 | 1.69 | 114.2330 | 22.6323 |
| 5.36 | 114.1483 | 22.5496 | 3.45 | 114.2263 | 22.6589 |
| 11.11 | 114.1176 | 22.6343 | 9.09 | 114.2211 | 22.6812 |
| 3.64 | 114.1096 | 22.6218 | 3.7 | 114.2234 | 22.6317 |
| 3.77 | 114.1097 | 22.6218 | 1.75 | 114.2163 | 22.6503 |
| 3.64 | 114.1193 | 22.6202 | 6.58 | 113.9263 | 22.5171 |
| 3.64 | 114.1115 | 22.6243 | 5.45 | 113.9445 | 22.5234 |
| 8.93 | 114.1097 | 22.6249 | 7.69 | 113.9496 | 22.5491 |
| 10.87 | 114.0623 | 22.5608 | 5.77 | 113.9279 | 22.5114 |
| 8.62 | 114.0587 | 22.5536 | 7.55 | 113.9358 | 22.5084 |
| 9.05 | 114.0650 | 22.5475 | 7.02 | 113.9351 | 22.5023 |
| 9.36 | 114.0661 | 22.5580 | 5.66 | 113.9210 | 22.5029 |
| 8.92 | 114.0666 | 22.5436 | 3.7 | 113.8916 | 22.4799 |
| 13.79 | 114.0652 | 22.5612 | 3.7 | 113.9270 | 22.4963 |
| 13.57 | 114.1776 | 22.5606 | 7.55 | 113.8842 | 22.4852 |
| 13.64 | 114.1730 | 22.5622 | 5.45 | 113.9312 | 22.4961 |
| 14.55 | 114.1792 | 22.5667 | 16.07 | 114.3261 | 22.6714 |
| 12.77 | 114.1645 | 22.5653 | 14.53 | 114.4757 | 22.5961 |
| 12.9 | 114.1625 | 22.5632 | 15.15 | 114.4801 | 22.5979 |
| 13.25 | 114.1855 | 22.5619 | 10.64 | 114.4648 | 22.5987 |
| 5.26 | 114.2376 | 22.7056 | 4.62 | 114.4952 | 22.5651 |
| 5.17 | 114.2421 | 22.7357 | 12.39 | 114.4849 | 22.5928 |
| 5.08 | 114.2313 | 22.7031 | 11.75 | 114.3797 | 22.7400 |
| 9.09 | 114.2510 | 22.6943 | 8.93 | 114.3988 | 22.7432 |
| 5.08 | 114.2481 | 22.6910 | 20 | 114.3688 | 22.7391 |
| 10.34 | 114.2485 | 22.7370 | 15.51 | 114.3572 | 22.6886 |
| 3.45 | 114.2692 | 22.7091 | 15.57 | 114.3565 | 22.7047 |
| 3.33 | 114.2572 | 22.7156 | 3.45 | 114.4090 | 22.7517 |
| 3.33 | 114.2703 | 22.7132 | 15.38 | 114.4088 | 22.6379 |
| 8.33 | 114.2655 | 22.7342 | 11.85 | 114.4167 | 22.6276 |
| 3.39 | 114.2307 | 22.7514 | 12.29 | 114.4220 | 22.6322 |
| 3.33 | 114.2374 | 22.7415 | 10.14 | 114.4234 | 22.6205 |
| 17.54 | 114.0317 | 22.6528 | 13.28 | 114.4168 | 22.6379 |
| 5 | 114.0171 | 22.6573 | 13.64 | 114.3629 | 22.6130 |
| 6.67 | 114.0182 | 22.6574 | 21.43 | 114.3658 | 22.7551 |
| 3.45 | 114.0326 | 22.6555 | 3.51 | 114.4024 | 22.7087 |
| 7.14 | 114.0331 | 22.6562 | 14.51 | 114.3692 | 22.7467 |
| 7.27 | 114.0376 | 22.6600 | 20.69 | 114.3721 | 22.6968 |
| 8.77 | 114.1025 | 22.5467 | 7.02 | 114.4005 | 22.7103 |
| 3.45 | 113.9072 | 22.7596 | 1.82 | 114.3410 | 22.6717 |
| 7.41 | 113.9074 | 22.7597 | 12.96 | 114.3456 | 22.6744 |
| 6.9 | 113.8885 | 22.7668 | 3.64 | 114.3342 | 22.6761 |
| 9.43 | 113.8885 | 22.7677 | 1.82 | 114.3325 | 22.6761 |
| 5 | 113.8756 | 22.7693 | 8.93 | 114.3358 | 22.6790 |
| 7.14 | 113.8758 | 22.7694 | 3.64 | 114.3402 | 22.6727 |
| 18.64 | 114.0689 | 22.5722 | 7.46 | 114.4895 | 22.5351 |
| 11.67 | 114.0632 | 22.5744 | 7.05 | 114.5452 | 22.5533 |
| 12.58 | 114.0412 | 22.5708 | 4.62 | 114.4979 | 22.5363 |
| 6.67 | 114.0350 | 22.5668 | 6.28 | 114.5161 | 22.5310 |
| 3.45 | 114.0474 | 22.5644 | 6.06 | 114.5150 | 22.5394 |
| 11.78 | 114.0744 | 22.5697 | 4.41 | 114.4889 | 22.5344 |
| 3.64 | 114.3005 | 22.6119 | 10.71 | 114.3290 | 22.6859 |
| 7.55 | 114.2964 | 22.6157 | 17.72 | 114.3314 | 22.6908 |
| 0.58 | 114.3048 | 22.6014 | 3.51 | 114.3248 | 22.6927 |
| 1.85 | 114.3044 | 22.6015 | 11.79 | 114.3443 | 22.6889 |
| 1.75 | 114.2975 | 22.6013 | 19.72 | 114.3511 | 22.6872 |
| 2.15 | 114.2975 | 22.6012 | 23.35 | 114.3462 | 22.6891 |
| 7.02 | 114.0387 | 22.5972 | 11.79 | 114.3777 | 22.6922 |
| 8.47 | 114.0371 | 22.5969 | 9.09 | 114.3281 | 22.6823 |
| 17.54 | 114.0205 | 22.6244 | 9.97 | 114.4045 | 22.6909 |
| 5.45 | 114.0307 | 22.6032 | 11.72 | 114.4054 | 22.6916 |
| 15.79 | 114.0219 | 22.6350 |  |  |  |

**Table 2. Original data of social and economic factors**

| **id** | **Name** | **Lar_hou** | **Gender** | **Kid** | **Teenager** | **Elderly** | **One_hou** | **Low_edu** | **Flow** | **Small_hou** | **Low_inc** | **Agri_fish** | **High_den** |
| --- | --- | --- | --- | --- | --- | --- | --- | --- | --- | --- | --- | --- | --- |
| 1 | Nanao | 2.7 | 134.32 | 6.65 | 11.53 | 6.71 | 30.24 | 20.3 | 28.33 | 13.93 | 21.83 | 0.94 | 145.51 |
| 2 | Dapeng | 2.32 | 146.04 | 5.82 | 9.6 | 3.91 | 39.97 | 11.27 | 47.38 | 23.32 | 21.83 | 0.94 | 743.21 |
| 3 | Kuiyong | 2.17 | 130.75 | 5.65 | 10.17 | 4.28 | 43.72 | 12.04 | 50.32 | 25.23 | 21.83 | 0.94 | 804.18 |
| 4 | Shijin | 2 | 175.28 | 3.71 | 6.57 | 2.25 | 48.02 | 13.7 | 53.75 | 37.61 | 14.61 | 0.38 | 1255.98 |
| 5 | Longtian | 1.94 | 138.43 | 5.26 | 8.67 | 1.93 | 49.15 | 9.76 | 62.59 | 34.51 | 14.61 | 0.38 | 3737.61 |
| 6 | Pingdi | 1.94 | 138.35 | 4.76 | 8.94 | 2.25 | 51.23 | 11.82 | 61.73 | 36.79 | 11.99 | 0.11 | 4130.9 |
| 7 | Kengzi | 2.15 | 134.77 | 5.8 | 10.24 | 2.33 | 44.36 | 11.19 | 57.85 | 36.53 | 14.61 | 0.38 | 5430.87 |
| 8 | Longgang | 2.32 | 116.9 | 6.63 | 11.31 | 2.91 | 40.17 | 8.66 | 44.2 | 23.92 | 11.99 | 0.11 | 5074.42 |
| 9 | Donghu | 2.86 | 103.43 | 6.48 | 12.94 | 5.97 | 23.04 | 7.66 | 26.46 | 12.65 | 20.64 | 0.08 | 3826.86 |
| 10 | Baolong | 2.24 | 133.63 | 5.61 | 9.57 | 2.26 | 40.66 | 9.35 | 56.03 | 27.63 | 11.99 | 0.11 | 9747.86 |
| 11 | Longcheng | 2.61 | 108.93 | 7.36 | 12.69 | 4.29 | 31.91 | 7.29 | 32.58 | 17.58 | 11.99 | 0.11 | 13869.95 |
| 12 | Pingshan | 2.49 | 121.87 | 7.15 | 12.78 | 3.15 | 35.08 | 10.41 | 44.37 | 29.42 | 14.61 | 0.38 | 11256.3 |
| 13 | Maluan | 2.05 | 147.11 | 6.3 | 10.28 | 2.53 | 50.07 | 8.47 | 55.05 | 29.8 | 14.61 | 0.38 | 2153.25 |
| 14 | Shatoujiao | 2.84 | 106.95 | 5.71 | 10.82 | 5.89 | 25.03 | 7.79 | 24.19 | 18 | 30.37 | 0.03 | 6561.64 |
| 15 | Xiangmihu | 2.88 | 101.2 | 5.9 | 11.31 | 7.6 | 21.59 | 4.84 | 19.4 | 5.39 | 30.76 | 0.02 | 16680.59 |
| 16 | Guanlan | 1.97 | 142.85 | 4.2 | 7.51 | 1.96 | 46.56 | 8.23 | 64.26 | 38.9 | 9.85 | 0.08 | 6393.16 |
| 17 | Nanwan | 2.36 | 115.18 | 6.82 | 10.71 | 3.45 | 38.72 | 8.17 | 38.41 | 21.66 | 11.99 | 0.11 | 15014.45 |
| 18 | Haishan | 2.88 | 115.09 | 5.72 | 10.26 | 5.83 | 23.34 | 5.45 | 28.3 | 15.94 | 30.37 | 0.03 | 8638.32 |
| 19 | Nanhu | 2.05 | 94.12 | 4.43 | 7.88 | 3.97 | 48.92 | 5.96 | 42.13 | 22.02 | 20.64 | 0.08 | 34923.86 |
| 20 | Huangbei | 2.73 | 103.55 | 6.01 | 11.26 | 5.68 | 27.63 | 7.55 | 26.63 | 19.62 | 20.64 | 0.08 | 25621.36 |
| 21 | Meisha | 2.39 | 132.48 | 4.54 | 8.68 | 4.84 | 34.7 | 8.43 | 40.37 | 18.14 | 30.37 | 0.03 | 1053.62 |
| 22 | Dongxiao | 3.09 | 111.84 | 5.95 | 12.59 | 4.61 | 19.2 | 8.11 | 24 | 22.93 | 20.64 | 0.08 | 33018.12 |
| 23 | Jihua | 2.58 | 113.41 | 7.24 | 11.99 | 3.36 | 31.32 | 8.37 | 36.72 | 26.42 | 11.99 | 0.11 | 12341.46 |
| 24 | Guanhu | 2.21 | 134.22 | 5.88 | 9.79 | 2.35 | 41.57 | 7.15 | 54.77 | 31.27 | 9.85 | 0.08 | 9803.91 |
| 25 | Longhua | 2.12 | 134.6 | 5.59 | 8.69 | 1.98 | 45.75 | 4.98 | 52.24 | 34.14 | 9.85 | 0.08 | 35881.95 |
| 26 | Fucheng | 1.86 | 162.27 | 4.98 | 7.35 | 1.66 | 52.52 | 5.24 | 66.61 | 30.46 | 9.85 | 0.08 | 10996.88 |
| 27 | Xinhu | 1.96 | 160.15 | 4.27 | 7.39 | 1.82 | 46.56 | 11.05 | 66.79 | 35 | 10.29 | 0.3 | 2810.59 |
| 28 | Guangming | 2.53 | 129.13 | 6.35 | 10.77 | 4.29 | 32.85 | 9.77 | 41.98 | 20.56 | 10.29 | 0.3 | 3692.51 |
| 29 | Matian | 2.02 | 134.17 | 5.84 | 9.62 | 1.92 | 46.3 | 9.05 | 61.8 | 33.35 | 10.29 | 0.3 | 17163.49 |
| 30 | Gongming | 2.02 | 132.39 | 5.25 | 9 | 1.84 | 46.21 | 9.6 | 65.58 | 33.22 | 10.29 | 0.3 | 8286.61 |
| 31 | Yanluo | 1.81 | 151.85 | 3.31 | 5.83 | 1.31 | 50.17 | 7.64 | 74.6 | 46.16 | 8.59 | 0.12 | 5502.02 |
| 32 | Songgang | 2.08 | 132.5 | 5.09 | 9.12 | 2.03 | 43.48 | 8.47 | 64.37 | 34.05 | 8.59 | 0.12 | 13130.61 |
| 33 | Liantang | 2.55 | 100.6 | 5.76 | 11.09 | 5.96 | 29.18 | 5.53 | 31.06 | 20.47 | 20.64 | 0.08 | 10638.93 |
| 34 | Futian | 2.44 | 107.86 | 5.93 | 9.88 | 3.85 | 37.78 | 7.23 | 31.94 | 24.61 | 30.76 | 0.02 | 34723.04 |
| 35 | Xinqiao | 2.08 | 132.68 | 5.2 | 8.54 | 1.7 | 42.48 | 7.86 | 61.44 | 34.53 | 8.59 | 0.12 | 13765.2 |
| 36 | Hangcheng | 2.02 | 137.36 | 4.94 | 7.96 | 1.73 | 44.76 | 7 | 59.03 | 46.11 | 8.59 | 0.12 | 6774.1 |
| 37 | Fuyong | 2.06 | 130.3 | 5.38 | 8.82 | 1.91 | 44.12 | 6.97 | 57.77 | 29.43 | 8.59 | 0.12 | 9262.89 |
| 38 | Yutang | 1.8 | 146.08 | 3.79 | 6.08 | 1.12 | 49.81 | 8.1 | 74.21 | 53.39 | 10.29 | 0.3 | 10472.82 |
| 39 | Dalang | 2.08 | 131.93 | 5.47 | 8.98 | 1.79 | 44.68 | 6.04 | 58.65 | 39.52 | 9.85 | 0.08 | 12150.82 |
| 40 | Fenghuang | 1.93 | 156.69 | 4.73 | 7.33 | 1.56 | 47.67 | 10.06 | 68.46 | 37.89 | 10.29 | 0.3 | 7283.44 |
| 41 | Shiyan | 1.99 | 138.91 | 4.89 | 8.02 | 1.83 | 45.75 | 5.98 | 65.27 | 40.58 | 8.59 | 0.12 | 7834.3 |
| 42 | Xili | 2.18 | 127.14 | 4.75 | 7.88 | 2.6 | 42.43 | 5.38 | 45.57 | 28.69 | 36.35 | 0.12 | 6715.83 |
| 43 | Xinan | 2.44 | 111.7 | 6.2 | 11.27 | 3.91 | 35.91 | 6.47 | 32.92 | 25.64 | 8.59 | 0.12 | 23895.36 |
| 44 | Bantian | 2.08 | 120.29 | 6.48 | 9.65 | 2.47 | 47.68 | 5.45 | 46.3 | 31.99 | 11.99 | 0.11 | 20630.03 |
| 45 | Minzhi | 2.34 | 108.56 | 7.43 | 11.01 | 3.3 | 38.96 | 5.25 | 39.46 | 22.03 | 9.85 | 0.08 | 21236.26 |
| 46 | Shatou | 2.17 | 109.68 | 4.69 | 7.93 | 3.36 | 42.59 | 5.77 | 40.83 | 35.16 | 30.76 | 0.02 | 21140.6 |
| 47 | Yuehai | 2.63 | 111.29 | 5.04 | 9.73 | 5.46 | 27.94 | 3.79 | 26 | 6.83 | 36.35 | 0.12 | 18442.76 |
| 48 | Xixiang | 2.18 | 119.33 | 6.15 | 9.94 | 2.64 | 43.47 | 6.49 | 44.65 | 32.96 | 8.59 | 0.12 | 14398.53 |
| 49 | Nantou | 2.58 | 111.21 | 5.17 | 9.78 | 5.11 | 30.05 | 5.16 | 34.45 | 21 | 36.35 | 0.12 | 17785.34 |
| 50 | Zhaoshang | 2.71 | 111.3 | 5.29 | 10.13 | 6.46 | 23.97 | 4.26 | 26.46 | 6.6 | 36.35 | 0.12 | 8375.41 |
| 51 | Shekou | 2.6 | 106.61 | 5.83 | 10.6 | 5.04 | 28.14 | 6.86 | 32.66 | 23.41 | 36.35 | 0.12 | 12432.2 |
| 52 | Guiyuan | 2.43 | 98.55 | 4.52 | 8.59 | 7.01 | 36.52 | 4.58 | 31.8 | 11.94 | 20.64 | 0.08 | 40823.46 |
| 53 | Nanshan | 2.52 | 111.23 | 5.57 | 9.61 | 4.23 | 31.19 | 5.75 | 39.29 | 21.79 | 36.35 | 0.12 | 13076.71 |
| 54 | Qingshuihe | 2.61 | 118.07 | 5.42 | 10.15 | 4.54 | 28.07 | 8.68 | 38.92 | 27.96 | 20.64 | 0.08 | 8851.26 |
| 55 | Lianhua | 2.66 | 98.47 | 5.46 | 10.62 | 7.93 | 26.91 | 4.77 | 23.72 | 10.87 | 30.76 | 0.02 | 23487.83 |
| 56 | Meilin | 2.56 | 104.25 | 5.52 | 10.08 | 5.68 | 30.3 | 5.71 | 29.05 | 19.55 | 30.76 | 0.02 | 11635.48 |
| 57 | Huafu | 2.7 | 113.35 | 5.29 | 10.68 | 6.29 | 27.77 | 5.32 | 25.33 | 13.37 | 30.76 | 0.02 | 10622.89 |
| 58 | Nanyuan | 2.77 | 124.32 | 6.07 | 10.79 | 3.34 | 29.36 | 7.67 | 34.19 | 35.29 | 30.76 | 0.02 | 56844.43 |
| 59 | Huaqiangbei | 2.63 | 133.93 | 5.35 | 9.79 | 5.8 | 30.27 | 4.89 | 28.7 | 18.36 | 30.76 | 0.02 | 22653.6 |
| 60 | Yuanling | 2.69 | 106.9 | 5.3 | 11.6 | 7.82 | 26.53 | 4.93 | 26.8 | 11.02 | 30.76 | 0.02 | 33627.26 |
| 61 | Buji | 2.66 | 104.14 | 7.27 | 12.37 | 4.48 | 29.93 | 7.44 | 29.43 | 20.58 | 11.99 | 0.11 | 34347.26 |
| 62 | Cuihu | 2.65 | 99.24 | 5.78 | 11.08 | 6.53 | 28.5 | 6.15 | 25.77 | 14.51 | 20.64 | 0.08 | 39033.79 |
| 63 | Pinghu | 2.21 | 129.51 | 5.12 | 8.89 | 2.13 | 39.74 | 9.82 | 54.91 | 38.02 | 11.99 | 0.11 | 10110.96 |
| 64 | Henggang | 2.29 | 115.96 | 6.51 | 10.74 | 2.72 | 38.75 | 8.07 | 43.33 | 33.65 | 11.99 | 0.11 | 12147.36 |
| 65 | Dongmeng | 2.71 | 102.76 | 5.36 | 10.85 | 5.01 | 30.32 | 9.35 | 28.34 | 32.98 | 20.64 | 0.08 | 43291.07 |
| 66 | Shajin | 2.01 | 133.9 | 4.7 | 7.86 | 1.78 | 43.65 | 6.77 | 66.23 | 37.71 | 8.59 | 0.12 | 15134.49 |
| 67 | Fuhai | 1.92 | 139.16 | 4.08 | 6.98 | 1.32 | 47 | 5.24 | 65.32 | 40.51 | 8.59 | 0.12 | 13942.01 |
| 68 | Fubao | 2.62 | 99.02 | 5.75 | 11.03 | 6.68 | 27.68 | 4.62 | 25.66 | 11.54 | 30.76 | 0.02 | 25083.13 |
| 69 | Shahe | 2.68 | 106.61 | 4.76 | 8.87 | 6.41 | 25.83 | 5.68 | 32.15 | 14.93 | 36.35 | 0.12 | 10127.9 |
| 70 | Taoyuan | 2.41 | 118.77 | 5.8 | 9.42 | 3.99 | 36.02 | 4.94 | 35.75 | 18.71 | 36.35 | 0.12 | 6255.33 |
| 71 | Yantian | 2.79 | 125.25 | 5.63 | 10.79 | 3.27 | 24.95 | 6.96 | 39.95 | 30.38 | 30.37 | 0.03 | 2033.67 |
| 72 | Sungang | 2.45 | 108.65 | 6.01 | 10.48 | 4.76 | 33.44 | 7.21 | 30.28 | 27.59 | 20.64 | 0.08 | 26427.64 |
| 73 | Biling | 2.14 | 137.31 | 5.55 | 10.05 | 2.64 | 44.66 | 13.28 | 56.98 | 35.35 | 14.61 | 0.38 | 2440.76 |
| 74 | Yuanshan | 2.11 | 134.06 | 4.78 | 9.08 | 1.97 | 43.18 | 9.13 | 57.16 | 36.91 | 11.99 | 0.11 | 5306.42 |
